# Pancreatic adenocarcinoma associated immune-gene signature as a novo risk factor for clinical prognosis prediction in hepatocellular carcinoma

**DOI:** 10.1038/s41598-022-16155-w

**Published:** 2022-07-13

**Authors:** Lei Dai, Joseph Mugaanyi, Xingchen Cai, Caide Lu, Changjiang Lu

**Affiliations:** Department of Hepatopancreatobiliary Surgery, Ningbo Medical Centre Lihuili Hospital, Ningbo University, 1111 Jiangnan Road, Ningbo, 315040 Zhejiang China

**Keywords:** Cancer, Computational biology and bioinformatics, Genetics, Immunology, Oncology

## Abstract

Pancreatic adenocarcinoma (PAAD) has high mortality and a very poor prognosis. Both surgery and chemotherapy have a suboptimal therapeutic effect, and this caused a need to find new approaches such as immunotherapy. Therefore, it is essential to develop a new model to predict patient prognosis and facilitate early intervention. Our study screened out and validated the target molecules based on the TCGA-PAAD dataset. We established the risk signature using univariate and multivariate Cox regression analysis and used GSE62452 and GSE28735 to verify the accuracy and reliability of the model. Expanded application of PAAD-immune-related genes signature (-IRGS) on other datasets was conducted, and the corresponding nomograms were constructed. We also analyzed the correlation between immune-related cells/genes and potential treatments. Our research demonstrated that a high riskscore of PAAD-IRGS in patients with PAAD was correlated with poor overall survival, disease-specific survival and progression free interval. The same results were observed in patients with LIHC. The models constructed were confirmed to be accurate and reliable. We found various correlations between PAAD-IRGS and immune-related cells/genes, and the potential therapeutic agents. These findings indicate that PAAD-IRGS may be a promising indicator for prognosis and of the tumor-immune microenvironment status in PAAD.

## Introduction

Pancreatic adenocarcinoma (PAAD) is one of the most common carcinomas globally and ranks 6th in cancer-related deaths^1^. Although considerable progress has been made in diagnosis and treatment^2^, the 5-year survival rate of PAAD is still less than 10%^3^. Therefore, there is still a need for new ways to predict patient prognosis and augment early intervention to maximize long-term survival.

The development of high-throughput sequencing has revolutionized DNA and RNA research^4^ and broadened the scope of research into potential biological progress and mechanisms of human disease^5^. Several studies have revealed differentially expressed mRNA/miRNA/lncRNA and differentially expressed genes (DEGs) of pancreatic carcinoma in recent years^6–10^. Although its theoretical value to the diagnosis and prognosis of pancreatic carcinoma has been detailed, the biological mechanisms, clinical significance, and the interaction between DEGs during pancreatic carcinoma tumorigenesis are yet to be explored.

Inflammation mediates and participates in various pathophysiological processes, including classic pathways of infection, immune elimination, tissue repair and regeneration^11,12^. The current studies put forwards a new point of view that inflammation is tightly associated with tumorigenesis, progression and metastasis of cancer^13,14^. Tumor risk factors can stimulate an extrinsic inflammatory response, while innate inflammatory response contributes to tumor progression, indicating that a complex network exists in tumor-immune microenvironment. Furthermore, immune-related genes (IRGs), including interleukin (IL)-10^15^, IL-6^16^, tumor necrosis factor-α (TNF-α)^17^ and (C-X-C motif) ligand (CXCL) chemokine family^18^ played a vital role in tumor proliferation, metabolism and metastasis. The occurrence and development of pancreatic cancer are recognized to be closely linked with inflammation. Local and systemic chronic inflammation could elevate the risk of PAAD, and PAAD-related inflammatory infiltration might simultaneously enhance tumor progression and metastasis^19^. Beyond the mechanism of an imbalance between inflammatory cell infiltration and immunosuppressive phenotype in the tumor-immunity microenvironment, obesity and diabetes are associated with promoting inflammation and inhibiting autophagy to Create a suitable environment for the tumorigenesis of PAAD through oxidative stress and metabolic impairments^20^.

Due to the interaction between immune-mediated inflammation and tumorigenesis, identifying whether immune response influences the prognosis of cancer patients has become a research hotspot. Quite a few carcinoma prognosis-related biomarkers have been identified and used to create models to predict patient survival^21–24^. However, there has not been much regarding IRGs signature for PAAD, let alone an immune-related prognostic model. In this study, we used the Cancer Genome Atlas (TCGA) and the Gene Expression Omnibus (GEO) database to screen out high-risk IRGs and create a novel risk-score signature and nomogram based on the IRGs for predicting the prognosis of PAAD patients. We also identified and comprehensively analyzed potential clinical therapeutic targets. Our findings may highlight the outstanding function of the IRGs signature in predicting PAAD patients’ prognosis and reveal its potential ability to predict the prognosis of patients with liver hepatocellular carcinoma (LIHC).

## Materials and methods

### Data acquisition and processing

We downloaded the TCGA-PAAD and TCGA-LIHC data sets, including: RNA sequences, raw clinical data and prognostic information, from the TCGA database (https://portal.gdc.cancer.gov/). Data of normal tissues from the GTEx database (https://gtexportal.org/) was obtained for supplementary. The gene expression data were converted to Transcripts per million reads (TPM) format and log2 transformed. Other data was cleaned and batch corrected with clinical information retained. Gene expression profiles and prognostic data of GSE28735^25^ and GSE62452^26^ were collected from the GEO database (http://www.ncbi.nlm.nih.gov/geo/) and used as validation datasets.

We obtained complete IRGs names, totaling 2483 from the “Resources-Gene Lists” module of the Immunology Database and Analysis Portal (ImmPort)^27^ (https://www.immport.org/home).

### DEGs & IRGs screening and intersecting

We first conducted a differential gene expression analysis to screen for genes expressed differently between pancreatic tumors and normal tissues, based on the RNA sequence dataset of TCGA-GTEx-PAAD. The log2(Fold Change) (FC) and adjusted p-value (*P*.adj) were calculated using R. Then, |log2(FC)|> 1 & *P*.adj < 0.05 was considered as the cut-off criteria for significant DEGs. These were subsequently intersected with the IRGs above. “ggplot2” package of R was used to visualize the performance with volcanoes plot and Venn diagram.

### Enrichment analysis for DEGs & IRGs

We performed the Kyoto encyclopedia of genes and genomes (KEGG) pathway and Gene Ontology (GO) enrichment analysis and the results were plotted using “ggplot2” (version 3.3.3) and “clusterProfiler” (version 3.14.3) packages in R^28^ for the genes of intersection. The settings modes used were: biological process (BP), cellular component (CC) and molecular function (MF) with *P*.adj < 0.05 were considered statistically significant and output visualized cnetplots respectively.

### Construction of PAAD-related IRGs signature (PAAD-IRGS) for prognosis

Based on the gene analysis above, we obtained independent immune-related prognostic risk genes using the Least absolute shrinkage and selection operator (Lasso) regression analysis^29^, followed by univariate and multivariate Cox regression analysis for further identification. LASSO is a popular algorithm, extensively utilized in medical studies^30–33^. Next, the Toil procedure from the university of California Santa Cruz (UCSC) Xena (^34^ was used to analyze the difference in the expression of the genes identified above in unpaired samples of PAAD. The log2(Transcripts per million (TPM) + 1) for log-scale was used in the assessments. The diagnostic value of these genes was evaluated using receiver operating characteristic (ROC) curves.

After this procedure, the optimal related IRGs were retained to establish the PAAD-IRGS. We compared the expression level of these genes in different pathologic stages and conducted the exclusively related KEGG and GO analysis. According to the expression level (EXP) and multivariate COX regression coefficient β value of the genes, the formula of the immune-related risk score signature is as follows^35^:

$$\mathrm{PAAD}-\mathrm{IRGS}=\sum_{k=1}^{n}EXPk*\mathrm{\beta k}.$$

Based on the risk score of each sample, the cohort was divided into two groups (low-risk with 0–50% vs high-risk with 50–100%). The performance of the classifier was assessed using ROC. Finally, we performed survival analyses of overall survival (OS) for single and combined genes using Kaplan–Meier and the log-rank test.

### Assessment of PAAD-IRGS and relevant clinical nomogram

The model to predict 1–3 years OS was evaluated using time-dependent ROC and decision curve analysis (DCA). Next, clinicopathologic characteristics of patients from TCGA-PAAD were collected and analyzed using univariate and multivariate COX regression analysis. Based on the clinical risk indicators (CRI) and PAAD-IRGS, we established a nomogram model to predict 1–3 years OS probability in PAAD patients. The nomogram was calibrated and assessed using DCA to verify its accuracy and reliability. The predictive accuracy of classical TMN-stage, PAAD-IRGS, CRI and nomogram were compared using the concordance index (C-Index).

### Validation and extended application of PAAD-IRGS

To validate the specificity and precision of PAAD-IRGS, we utilized GSE28735 and GSE62452, which contained sufficient gene expression and prognosis data, to conduct differential expression analysis, survival analysis, diagnostic/prognostic value and applicability of clinical decision evaluation.

For assessing the extended applicability of PAAD-IRGS, considering the disease categories and histological homologies, we selected the TCGA-LIHC (n = 374) for further validation of the model. The difference in expression level of these genes between tumor and normal tissues was compared, and their individual and unified diagnostic ability. According to the standard established above, the LIHC cohort was grouped as low- (0–50%) and high-risk (50–100%) groups. Single-gene and unified signature OS analyses were performed using Kaplan–Meier curves, followed by time-dependent ROC and DCA analysis. Similarly, we established a nomogram model to predict 1–3 years OS probability in LIHC patients, based on the PAAD-IRGS and CRI, obtained from the TCGA-LIHC cohort through univariate and multivariate COX regression analysis. Calibration and DCA were performed to verify the reliability and accuracy of the model. Then the classical TMN-stage, PAAD-IRGS, CRI of TCGA-LIHC, and synthetic nomogram were compared with C-Index to assess their accuracy and clinical value for LIHC.

Furthermore, we expanded the application of PAAD-IRGS to predict 1–3 years disease-specific survival (DSS) and progression-free interval (PFI) of patients in TCGA-PAAD.

### Immuno-correlation analysis and drug prediction of PAAD-IRGS

We conducted the PAAD-IRGS risk score correlation analysis with 24 immune-related cells^36^ in PAAD using the spearman’s test^37^. Subsequently, survival analysis of several significant immune-related cells was conducted to identify whether they were risk factors of PAAD using tumor immune estimation resource (TIMER), version 2.0 database^38–40^. Then we downloaded the immunophenoscore (IPS)^41^ data from The Cancer Immunome Atlas (TCIA) database (https://tcia.at/patients), which supports results of comprehensive immunogenomic analysis of next generation sequencing data (NGS) based on TCGA^42^, for analyzing the correlation between PAAD-IRGS and immune response in PAAD patients.

Relationships between PAAD-IRGS risk score and three kinds of immunomodulators expression in PAAD based on TCGA were explored and visualized with heatmaps, as well as relevant drug prediction accordingly via tumor-immune system interaction database (TISIDB)^43^ (http://cis.hku.hk/TISIDB/index.php), integrating multiple heterogeneous data. We searched the website with the gene symbol S100P, S100A2 and MMP12 and download relevant information in the "drug" module. Circle map and annotations were performed accordingly.

### Analysis of protein expression of the PAAD-IRGS

The human protein atlas (HPA) database^44^,a spatial map of the human proteome (http://www.proteinatlas.org/humanproteome/pathology) was used to ascertain the physiological and pathological expression data of S100P, S100A2 and MMP12. As supplementary, we used UALCAN (http://ualcan.path.uab.edu/index.html) to conduct protein level analysis of S100P, S100A2 and MMP12 genes. It is a comprehensive and interactive public resource for cancer OMICS data analysis^45^, provided by the Clinical proteomic tumor analysis consortium (CPTAC) dataset^46^.

### Statistical analysis

All statistical analyses were performed with R (version 3.6.3). Normally distributed variables were analyzed using the t-test and one-way ANOVA test and non-normally distributed variables with nonparametric tests. Log-rank test and Cox regression were used for survival analysis, Pearson’s correlation and spearman’s rank correlation test for correlation analysis. *P* or *P*.adj < 0.05 was considered statistically significant. The correlations was defined as follows: 0.00–0.10 (negligible), 0.10–0.39 (weak), 0.40–0.69 (moderate), 0.70–0.89 (strong), 0.90–1.00 (very strong)^47^.

## Results

**Figure 1:**
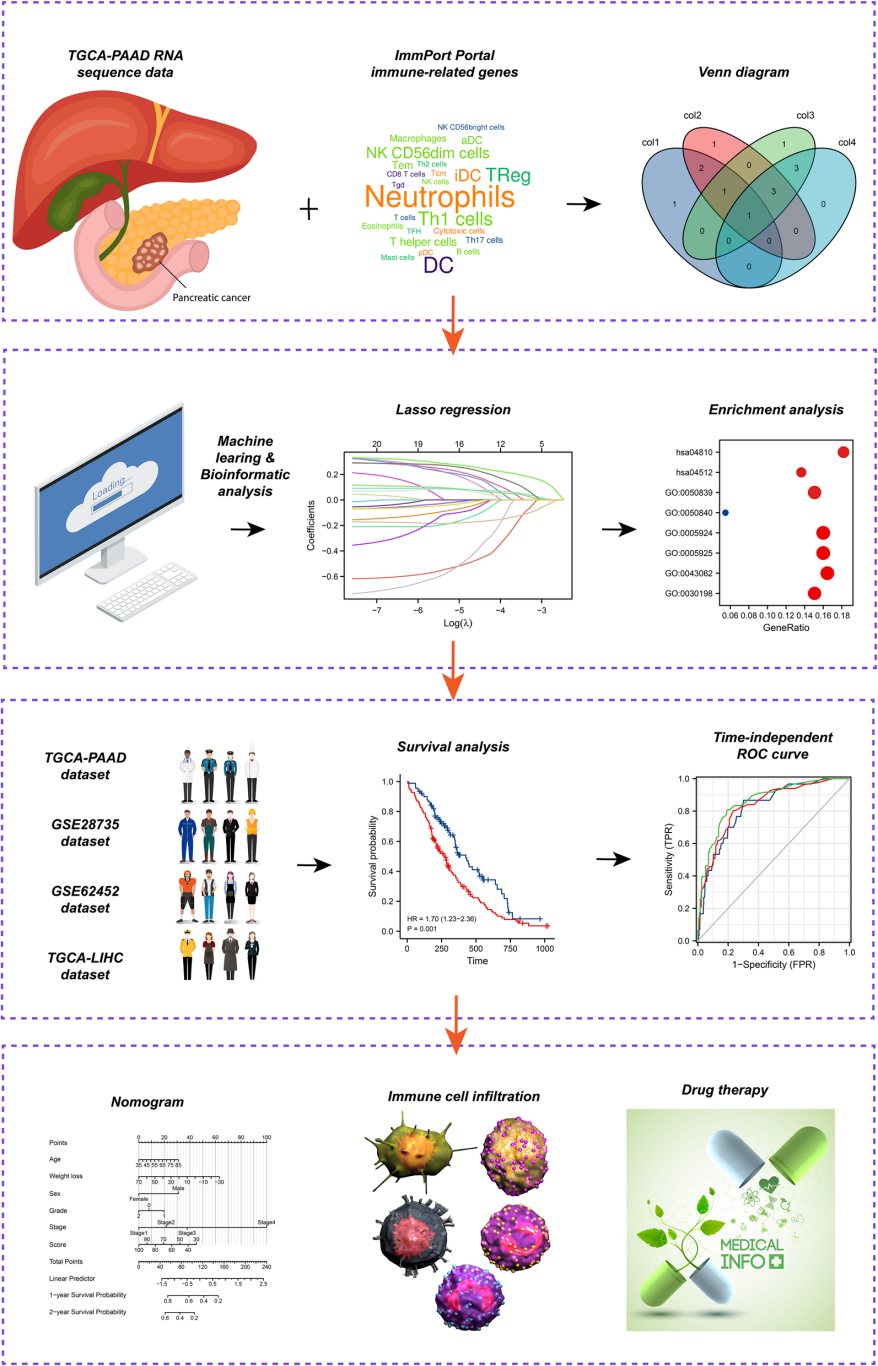
Study design flow chart. *TCGA* the cancer genome atlas, *PAAD* pancreatic adenocarcinoma, *LIHC* liver hepatocellular carcinoma, *ROC* receiver operating characteristic curve. This cover has been designed using images from Freepik.com.

The study design for this work is shown in Fig. 1.

### DEGs & IRGs analysis

**Figure 2:**
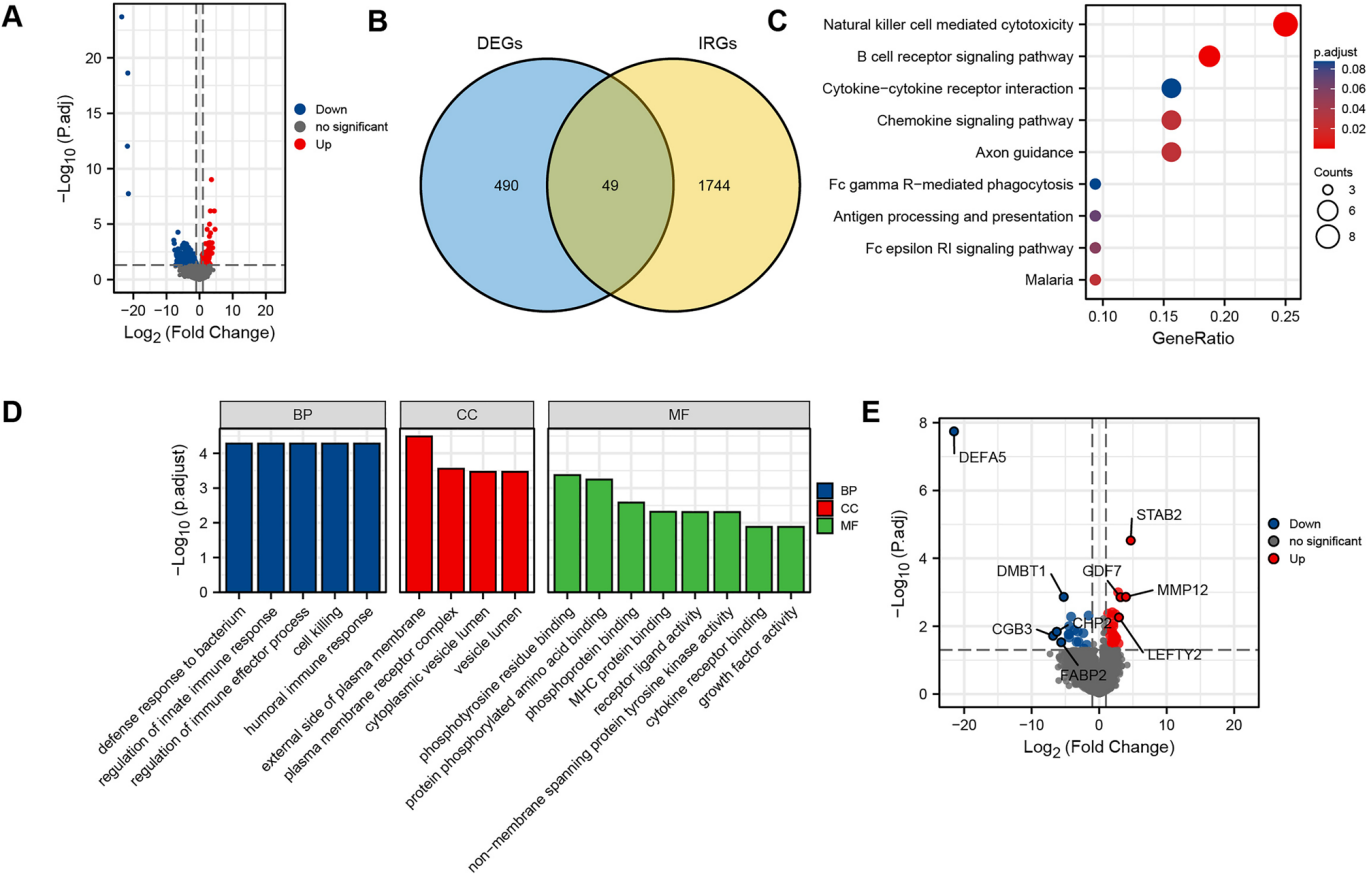
Screening of differentially expressed genes and immune-related genes related to pancreatic adenocarcinoma. (**A**) Volcano plot of 539 DEGs; (**B**) Venn diagram of intersection of DEGs and IRGs. (**C**) KEGG pathways analysis of genes in DEGs & IRGs; (**D**) GO analysis of genes in DEGs & IRGs; (**E**) Volcano plot of 49 genes in DEGs & IRGs. *DEGs* differentially expressed genes, *IRGs* immune-related genes, *KEGG* Kyoto Encyclopedia of Genes and Genomes, *GO* Gene Ontology.

178 PAAD patients with gene expression and prognostic information and 4 matched adjacent normal samples were included in the training cohort. 25,597 gene IDs were analyzed after removing null values, in which we obtained 539 differentially expressed genes that met the cut-off criterion of |log2(FC)|> 1 & *P*.adj < 0.05 in PAAD (236 genes up-regulated while 303 down-regulated) (Fig. 2A). Through the intersection of 490 DEGs and 1744 IRGs, 49 differentially expressed IRGs in PAAD were screened out (Fig. 2B).

### Enrichment analysis

The KEGG pathways which were most associated with immunity involved in natural killer mediated cytotoxicity (*P* < 0.001), B cell receptor signaling pathway (*P* < 0.001) and chemokine signaling pathway (*P* < 0.05) (Fig. 2C). Specifically, regulation of the immune effector process, cell killing and humoral immune response of the biological process (BP) module (all *P* < 0.001) were observed to be associated with immunity. So was major histocompatibility complex (MHC) protein binding and cytokine receptor binding of molecular functional (MF) module (Fig. 2D). Gene overlap is highlighted in the volcano plot (Fig. 2E).

### Construction and assessment of PAAD-IRGS

**Figure 3:**
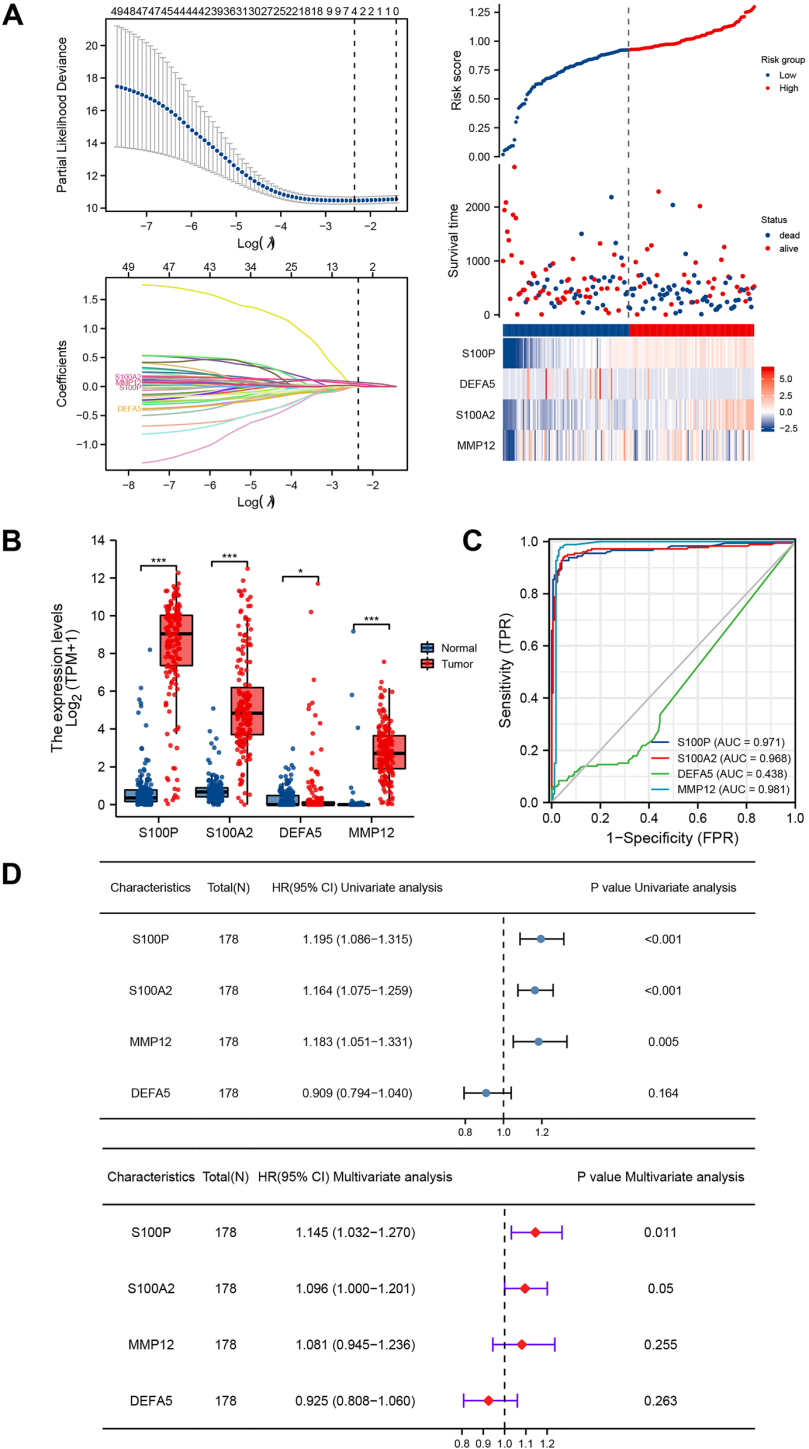
Establishment of IRGs signature (IRGS). (**A**) Ten-time cross-validation for tuning parameter selection in the Lasso regression model and risk analysis of four immune-related genes in patients with PAAD; (**B**) differential expression of four IRGs between tumor and normal tissues of patients with PAAD; (**C**) diagnostic value of four IRGs for patients with PAAD; (**D**) univariate and multivariate COX analysis of four IRGs is shown in forest map. *P < 0.05, **P < 0.01, ***P < 0.001.

We further analyzed the genes identified above to identify the potential diagnostic and prognostic value of IRGs in PAAD. Based on LASSO regression analysis, four prognostic risk biomarkers were identified (high expression of S100P, S100A2, and MMP12 was associated with poor prognosis, while low expression of DEFA5 was associated with better prognosis) (Fig. 3A). S100P, S100A2 and MMP12 were expressed higher in tumor tissues, compared with normal tissues (*P* < 0.001), while the opposite was true for DEFA5 expression (*P* < 0.05) (Fig. 3B). The area under curve (AUC) of S100P, S100A2, and MMP12 were 0.971, 0.968, and 0.981, indicating their excellent diagnostic value. However, DEFA5 was considered an inefficient biomarker for diagnosis (AUC = 0.438) (Fig. 3C). Subsequent univariate and multivariate COX regression analyses were conducted on the four genes, excluding DEFA5 (*P* = 0.164) (Fig. 3D). The model of PAAD-IRGS was finally comprised of S100P, S100A2 and MMP12. We plugged the corresponding regression coefficients into the equation as follows to complete the establishment of PAAD-IRGS: PAAD-IRGS = EXP(S100P) × 0.132 + EXP(S100A2) × 0.098 + EXP(MMP12) × 0.095.

**Figure 4:**
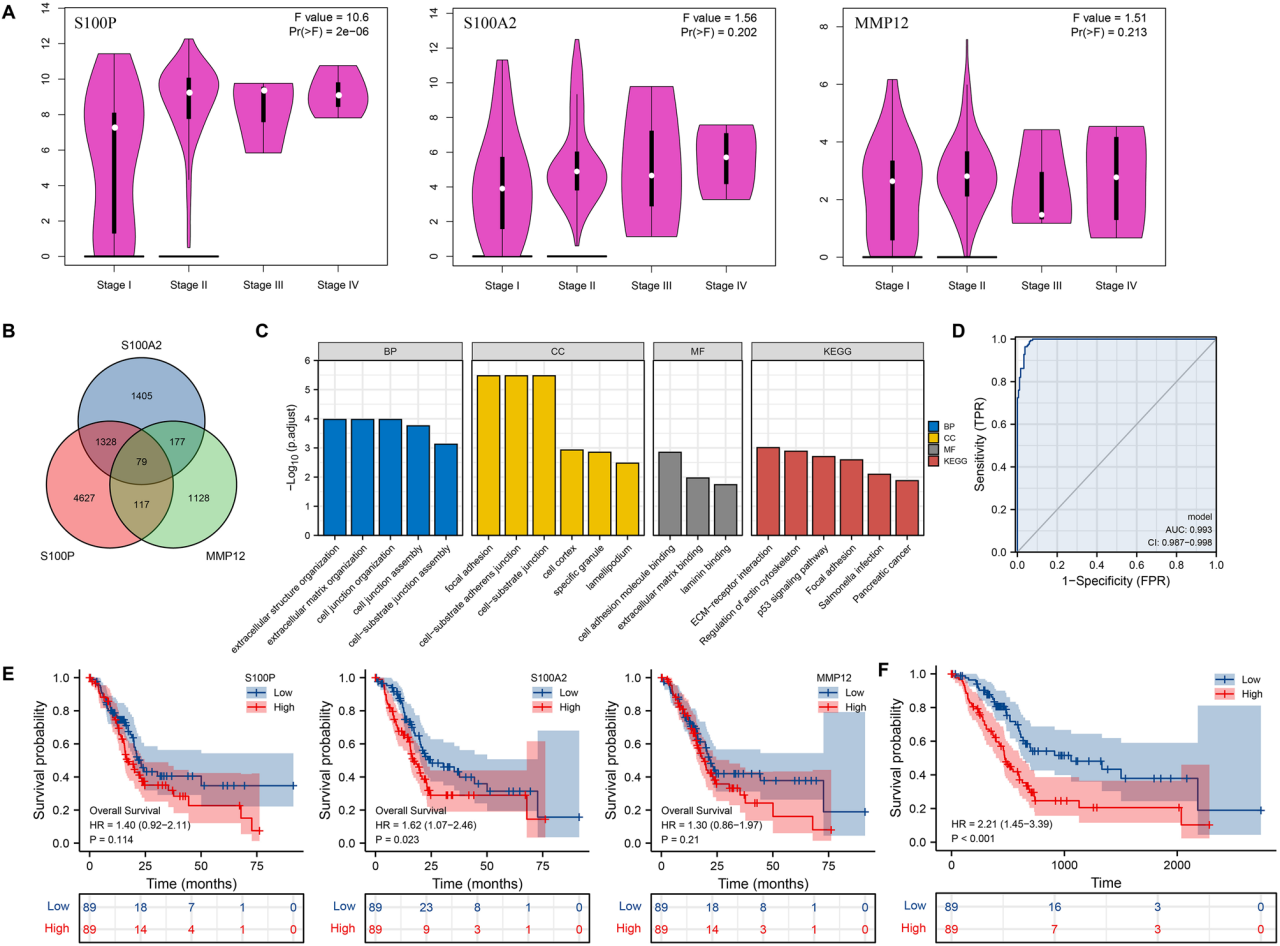
A comprehensive evaluation of IRGS. (**A**) Expression of 3 signature genes in different pathologic stages of PAAD; (**B**) Venn diagram of intersection of enrichment analysis of 3 signature genes; (**C**) GO and KEGG analysis of 3 signature genes; (**D**) diagnostic value of IRGS in PAAD; (**E**) single-gene survival analysis of OS was shown in Kaplan–Meier curves respectively; (**F**) Kaplan–Meier curves show that OS was significantly different between the low- and high-risk groups in TCGA-PAAD. *OS* overall survival; *P < 0.05, **P < 0.01, ***P < 0.001.

Furthermore, we performed PAAD-IRGS specialized differential expression analysis in different pathology stages. Using the Gene expression profiling interactive analysis (GEPIA) database^48^, a statistically significant difference in S100P expression was observed in different pathology stages of TCGA-PAAD (*P* < 0.001) (Fig. 4A). After a single gene correlation analysis of these three genes, we obtained 79 co-correlated genes (Fig. 4B). Based on further enrichment analysis, KEGG pathways seemingly involved in ECM-receptor interaction, regulation of actin cytoskeleton, p53 signaling pathway, focal adhesion and pancreatic cancer, and GO pathway focused on cell-membrane organization and connection (Fig. 4C).

The model showed a better diagnostic capability than individual genes with an AUC of 0.993 (95% confidence interval (CI) = 0.987–0.998) (Fig. 4D). By single gene survival analysis, we observed that patients in S100A2 high-expression group had a worse OS than patients in S100A2 low-expression group (hazard ratio (HR) = 1.62, 95% CI = 1.07–2.46, *P* = 0.023). However, there is no statistically significant difference between low and high expression groups of S100P or MMP12 (Fig. 4E). Patients in the PAAD-IRGS high-risk score group had a much worse OS than patients in low-risk score group (HR = 2.21, 95% CI = 1.45–3.39, *P* < 0.001) (Fig. 4F).

### Establishment of PAAD-IRGS based prognosis model

**Figure 5:**
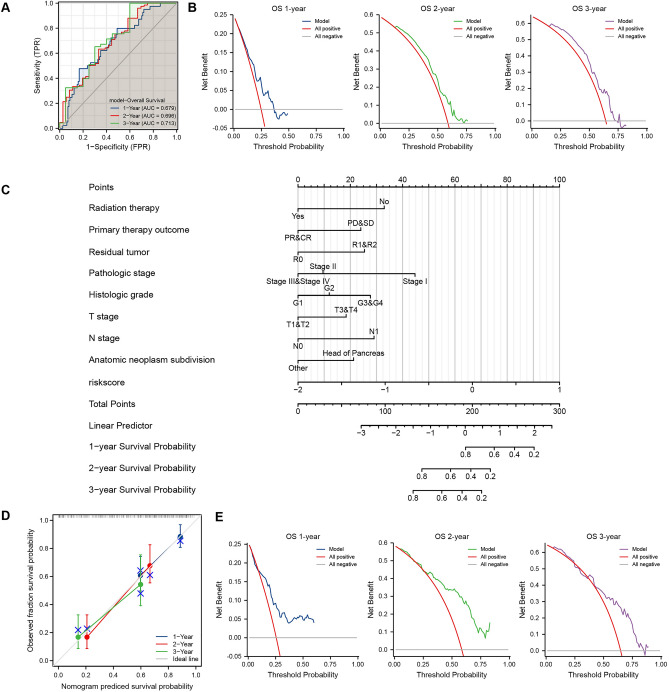
Evaluation of PAAD-IRGS and establishment and assessment of relevant nomograms. (**A**) The time-dependent ROC curve of the PAAD-IRGS for predicting 1, 2, and 3-year OS; (**B**) decision curve analysis for evaluating the PAAD-IRGS; (**C**) an PAAD-IRGS -based nomogram included with 8 clinical components predicting 1, 2, and 3-year OS of PAAD; (**D**) nomogram calibration curve for 1, 2, and 3-year. (**E**) decision curve analysis for evaluating the net benefits of nomogram at 1, 2, and 3 years. *P < 0.05, **P < 0.01, ***P < 0.001.

A total of 182 TCGA-PAAD patients were included in the prognostic analysis with the baseline characteristics shown in Table 1. Time-dependent ROC analysis was conducted to assess the accuracy of PAAD-IRGS for prediction of OS in PAAD patients. It showed an above average performance of 1 (AUC = 0.679), 2 (AUC = 0.696), and 3 years (AUC = 0.713) (Fig. 5A). DCA showed that model has a good clinical utility (Fig. 5B). T3&T4 stage (*P* = 0.030), N1 stage (*P* = 0.004), pathological stage II (*P* = 0.033), radiation therapy (*P* = 0.013), primary therapy outcome of PR&CR (*P* < 0.001), R1&R2 resection (*P* = 0.028), histological grade G2 (*P* = 0.047)/G3&G4 (*P* = 0.008), non-head of pancreas neoplasm (*P* = 0.004) and PAAD-IRGS (*P* < 0.001) were significantly correlated with OS. Radiation therapy (HR = 0.437, 95%CI = 0.228–0.835, *P* = 0.012), primary therapy outcome of PR&CR (HR = 0.547, 95%CI = 0.324–0.923, *P* = 0.024), R1&R2 resection (HR = 1.896, 95%CI = 1.087–3.308, *P* = 0.024) and PAAD-IRGS (HR = 2.312, 95%CI = 1.245–4.294,*P* = 0.008) were independent factors impacting the OS of patients with PAAD (Table 2). Based on the above analysis, the nomogram incorporating PAAD-IRGS and multiple clinicopathological characteristics was plotted (Fig. 5C). Through comparison, the concordance index (C-Index) of TNM-stage, PAAD-IRGS, Nomogram (only clinical indicators), and Nomogram + IRGS was 0.567, 0.639, 0.706 and 0.723 (Table 3), respectively. Additionally, Nomogram calibration curves (Fig. 5D) showed good predictive accuracy of the model and DCA (Fig. 5E).

**Table 1:** Baseline characteristics of patients (TCGA-PAAD).

Characteristics	Levels	Low-risk group	High-risk group	*p* value*
		N = 89	N = 89	
T stage, n (%)	T1	6 (6.9%)	1 (1.1%)	**0.037**
	T2	16 (18.4%)	8 (9%)	
	T3	64 (73.6%)	78 (87.6%)	
	T4	1 (1.1%)	2 (2.2%)	
N stage, n (%)	N0	27 (31.8%)	23 (26.1%)	0.517
	N1	58 (68.2%)	65 (73.9%)	
M stage, n (%)	M0	37 (97.4%)	42 (91.3%)	0.372
	M1	1 (2.6%)	4 (8.7%)	
Pathologic stage, n (%)	Stage I	16 (18.4%)	5 (5.7%)	**0.022**
	Stage II	69 (79.3%)	77 (87.5%)	
	Stage III	1 (1.1%)	2 (2.3%)	
	Stage IV	1 (1.1%)	4 (4.5%)	
Radiation therapy, n (%)	No	53 (65.4%)	65 (79.3%)	0.072
	Yes	28 (34.6%)	17 (20.7%)	
Primary therapy outcome, n (%)	PD	19 (28.8%)	30 (41.1%)	0.386
	SD	5 (7.6%)	4 (5.5%)	
	PR	4 (6.1%)	6 (8.2%)	
	CR	38 (57.6%)	33 (45.2%)	
Gender, n (%)	Female	40 (44.9%)	40 (44.9%)	1.000
	Male	49 (55.1%)	49 (55.1%)	
Age, n (%)	≤ 65	49 (55.1%)	44 (49.4%)	0.548
	> 65	40 (44.9%)	45 (50.6%)	
Residual tumor, n (%)	R0	59 (70.2%)	48 (60%)	0.201
	R1	24 (28.6%)	28 (35%)	
	R2	1 (1.2%)	4 (5%)	
Histologic grade, n (%)	G1	23 (26.4%)	8 (9%)	**0.001**
	G2	46 (52.9%)	49 (55.1%)	
	G3	16 (18.4%)	32 (36%)	
	G4	2 (2.3%)	0 (0%)	
Anatomic neoplasm subdivision, n (%)	Head of Pancreas	72 (80.9%)	66 (74.2%)	0.369
	Other	17 (19.1%)	23 (25.8%)	
Smoker, n (%)	No	30 (44.1%)	35 (46.1%)	0.948
	Yes	38 (55.9%)	41 (53.9%)	
Alcohol history, n (%)	No	36 (43.9%)	29 (34.5%)	0.281
	Yes	46 (56.1%)	55 (65.5%)	
History of diabetes, n (%)	No	48 (69.6%)	60 (77.9%)	0.337
	Yes	21 (30.4%)	17 (22.1%)	
History of chronic pancreatitis, n (%)	No	61 (92.4%)	67 (89.3%)	0.733
	Yes	5 (7.6%)	8 (10.7%)	
Family history of cancer, n (%)	No	21 (39.6%)	26 (45.6%)	0.659
	Yes	32 (60.4%)	31 (54.4%)	

Data are presented as n (%).

*PAAD* pancreatic adenocarcinoma.

*Compared with each group (Fisher exact test, or Pearson’s chi-square test). *p* value < 0.05 was considered statistically significant (highlighted in bold).

**Table 2:** The univariate and multivariate analysis for the OS (TCGA-PAAD).

Characteristics		Total(N)	Univariate analysis		Multivariate analysis	
			Hazard ratio (95% CI)	*P* value*	Hazard ratio (95% CI)	*P* value*
T stage	T1&T2	31	Reference		NA	
	T3&T4	145	2.023 (1.072–3.816)	**0.030**	1.588 (0.558–4.523)	0.386
N stage	N0	50	Reference		NA	
	N1	123	2.154 (1.282–3.618)	**0.004**	2.077 (0.952–4.529)	0.066
M stage	M0	79	Reference			
	M1	5	0.756 (0.181–3.157)	0.701		
Pathologic stage	Stage I	21	Reference		NA	
	Stage II	146	2.332 (1.069–5.088)	**0.033**	0.413 (0.091–1.868)	0.251
	Stage III&IV	8	1.446 (0.369–5.664)	0.597	0.325 (0.043–2.451)	0.275
Gender	Female	80	Reference			
	Male	98	0.809 (0.537–1.219)	0.311		
Age	≤ 65	93	Reference			
	> 65	85	1.290 (0.854–1.948)	0.227		
Radiation therapy	No	118	Reference		NA	
	Yes	45	0.508 (0.298–0.866)	**0.013**	0.437 (0.228–0.835)	**0.012**
Primary therapy outcome	PD&SD	58	Reference		NA	
	PR&CR	81	0.425 (0.267–0.677)	** < 0.001**	0.547 (0.324–0.923)	**0.024**
Residual tumor	R0	107	Reference		NA	
	R1&2	57	1.645 (1.056–2.561)	**0.028**	1.896 (1.087–3.308)	**0.024**
Histologic grade	G1	31	Reference		NA	
	G2	95	1.961 (1.008–3.812)	**0.047**	1.351 (0.523–3.488)	0.535
	G3&G4	50	2.578 (1.284–5.176)	**0.008**	2.005 (0.732–5.494)	0.176
Anatomic neoplasm subdivision	Head of Pancreas	138	Reference		NA	
	Other	40	0.417 (0.231–0.754)	**0.004**	0.586 (0.275–1.251)	0.167
Smoker	No	65	Reference			
	Yes	79	1.086 (0.687–1.719)	0.724		
Alcohol history	No	65	Reference			
	Yes	101	1.147 (0.738–1.783)	0.542		
History of diabetes	No	108	Reference			
	Yes	38	0.927 (0.532–1.615)	0.790		
History of chronic pancreatitis	No	128	Reference			
	Yes	13	1.177 (0.562–2.464)	0.666		
Family history of cancer	No	47	Reference			
	Yes	63	1.117 (0.650–1.920)	0.689		
Model		178	2.718 (1.733–4.263)	** < 0.001**	2.312 (1.245–4.294)	**0.008**

*OS* overall survival, *CI* confidence interval, *NA* reference group or could not be evaluated.

*Compared with each group (Log-Rank test or Omnibus test for univariate, Cox regression analysis with adjusted hazard for multivariate). *p* < 0.05 means statistically significant (highlighted in bold).

**Table 3:** The C-Index values of TNM-stage, PAAD-IRGS, nomogram and nomogram + IRGS in different cohorts.

Cohorts	C-Index (95% CI)			
	TNM-stage	PAAD-IRGS	Nomogram	Nomogram + IRGS
PAAD-OS	0.567 (0.538–0.596)	0.639 (0.609–0.670)	0.706 (0.672–0.740)	0.723 (0.690–0.756)
LIHC-OS	0.616 (0.588–0.644)	0.615 (0.588–0.644)	0.637 (0.603–0.672)	0.666 (0.630–0.701)
PAAD-DSS	0.571 (0.528–0.614)	0.680 (0.649–0.711)	0.749 (0.714–0.784)	0.775 (0.742–0.808)
PAAD-PFI	0.543 (0.508–0.578)	0.649 (0.618–0.681)	0.699 (0.664–0.733)	0.742 (0.712–0.771)

*C-Index* concordance index, *IRGS* immune-related genes signature, *PAAD* pancreatic adenocarcinoma, *LIHC* liver hepatocellular carcinoma, *OS* overall survival, *DSS* disease specific survival, *PFI* progress free interval.

### Validation and extension of PAAD-IRGS

**Figure 6:**
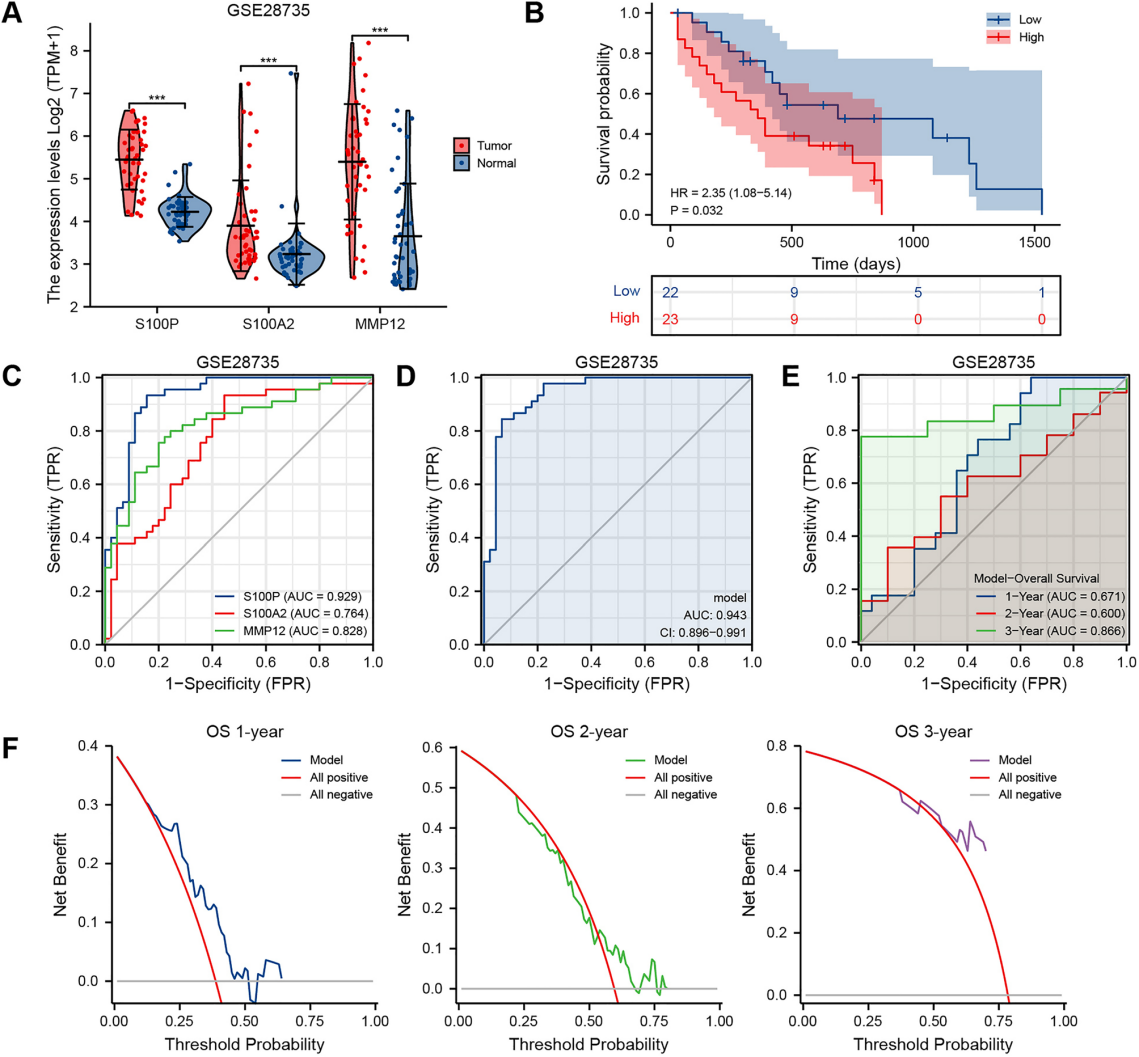
Validation of PAAD-IRGS with GSE28735. (**A**) Expression level of 3 IRGs in GSE28735 cohort; (**B**) Kaplan–Meier curves show a better OS in the low-risk group than the high-risk group; (**C**) diagnostic value of 3 IRGs for PAAD patients in GSE28735 cohort; (**D**) diagnostic value of PAAD-IRGS in GSE28735 cohort; (**E**) time-dependent ROC curve analysis of the PAAD-IRGS at 1, 2, and 3 years in GSE28735 cohort; (**F**) decision curve analysis for evaluating the net benefits of PAAD-IRGS in GSE28735 cohort. *P < 0.05, **P < 0.01, ***P < 0.001.

**Figure 7:**
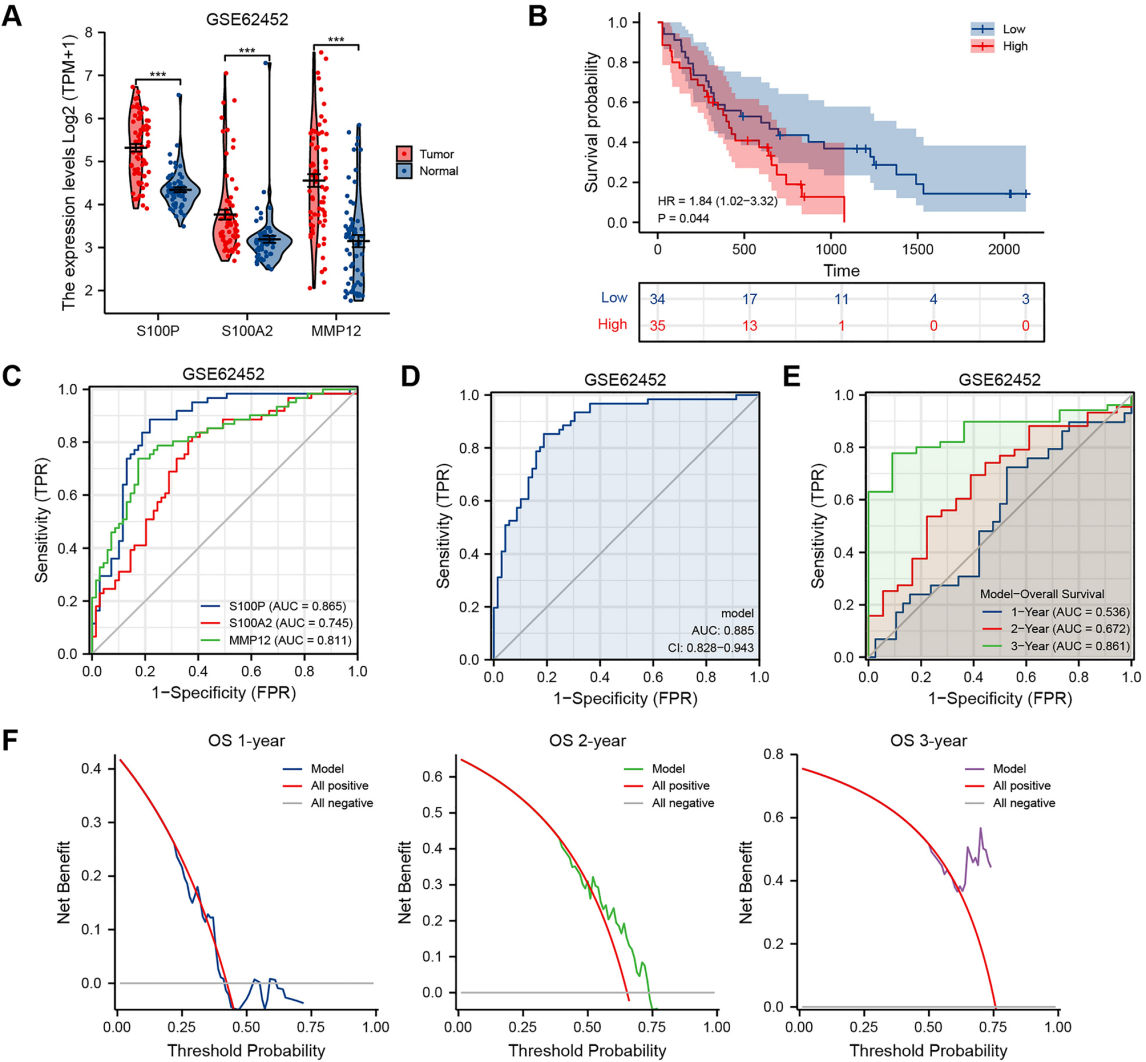
Validation of PAAD-IRGS with GSE62452. (**A**) Expression level of 3 IRGs in GSE62452 cohort; (**B**) Kaplan–Meier curves show a better OS in the low-risk group than the high-risk group; (**C**) diagnostic value of 3 IRGs for PAAD patients in GSE62452 cohort; (**D**) diagnostic value of PAAD-IRGS in GSE62452 cohort; (**E**) time-dependent ROC curve analysis of the PAAD-IRGS at 1, 2, and 3 years in GSE62452 cohort; (**F**) decision curve analysis for evaluating the net benefits of PAAD-IRGS in GSE62452 cohort. *P < 0.05, **P < 0.01, ***P < 0.001.

For further validation of the reliability of PAAD-IRGS, we employed two datasets of the GEO database. Differential expression, survival, diagnostic value, prognostic value analysis and DCA were performed in both datasets. The three genes had a higher expression in the tumor tissues than in normal tissues (*P* < 0.001) of GSE28735 (Fig. 6A). Patients in a high-risk group of PAAD-IRGS had worse OS than that of the low-risk group (HR = 2.35, 95%CI = 1.08–5.14, *P* = 0.032) (Fig. 6B). Consistent with the results above, although S100P (AUC = 0.929), S100A2 (AUC = 0.764), MMP12 (AUC = 0.828) (Fig. 6C) showed considerable diagnostic values for PAAD respectively, PAAD-IRGS had the optimum diagnostic ability (AUC = 0.943, 95%CI = 0.896–0.991) (Fig. 6D). In addition, time-dependent ROC showed the model had an above-average ability to predict 1—(AUC = 0.671), 2—(AUC = 0.600), and 3—year OSs (AUC = 0.866) (Fig. 6E). The model also had an acceptable net benefit based on DCA (C-Index = 0.644, 95%CI = 0.598–0.690) (Fig. 6F). Similar results of differential expression (*P* < 0.001) (Fig. 7A) and OS probability (HR = 1.84, 95%CI = 1.02–3.32, *P* = 0.044) (Fig. 7B) were obtained in GSE62452, as well as the independent diagnostic value of S100P (AUC = 0.865), S100A2 (AUC = 0.745), MMP12 (AUC = 0.811) (Fig. 7C) and all of them combined (AUC = 0.885, 95%CI = 0.828–0.943) (Fig. 7D). The corresponding ROC analysis showed an above-average performance in predicting 1—(AUC = 0.536), 2—(AUC = 0.672), and 3—year prognosis (AUC = 0.861) (Fig. 7E). Although 1-year net benefit of prognostic prediction was not satisfactory, 2- and 3-years showed a much better net benefit (C-Index = 0.580, 95%CI = 0.531–0.629) (Fig. 7F).

**Figure 8:**
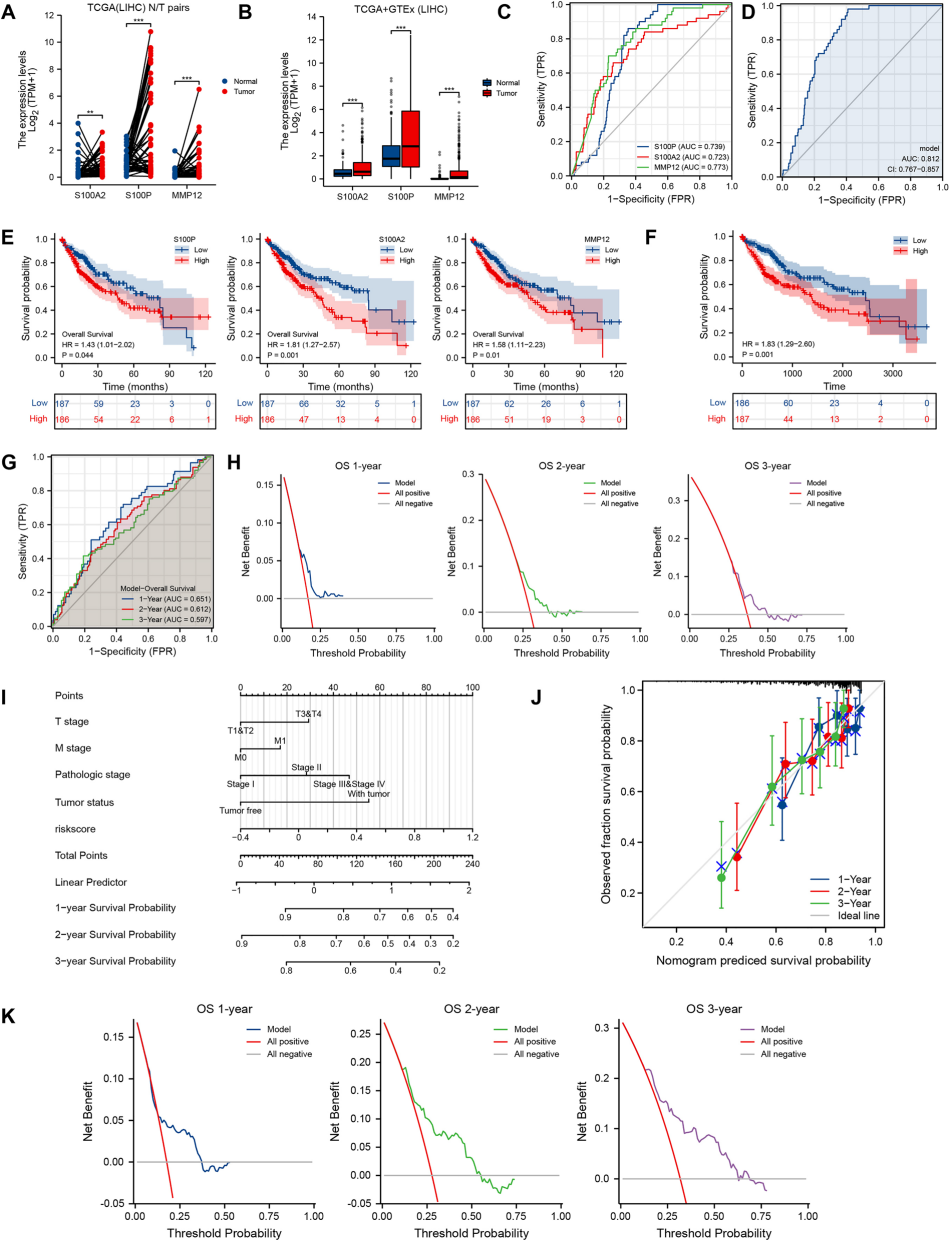
Validation of PAAD-IRGS with TCGA-LIHC. (**A**) Paired comparison of 3 IRGs expression levels in TCGA-LIHC; (**B**) unpaired comparison of 3 IRGs expression levels in TCGA-LIHC by including the relevant normal tissues of the GTEx database as controls; (**C**) diagnostic value of 3 IRGs for TCGA-LIHC patients; (**D**) diagnostic value of PAAD-IRGS for TCGA-LIHC patients; (**E**) single-gene survival analysis of OS for TCGA-LIHC; (**F**) PAAD-IRGS survival analysis of OS for TCGA-LIHC; (**G**) time-dependent ROC curve analysis of the PAAD-IRGS at 1, 2, and 3 years for TCGA-LIHC; (**H**) decision curve analysis for evaluating the net benefits of PAAD-IRGS for TCGA-LIHC; (**I**) an PAAD-IRGS-based nomogram included with 4 clinical components predicting 1, 2, and 3-year OS of TCGA-LIHC; (**J**) nomogram calibration curve for 1, 2, and 3-year. (**K**) Decision curve analysis for evaluating the net benefits of nomogram at 1, 2, and 3 years. *P < 0.05, **P < 0.01, ***P < 0.001.

Hepatobiliary and pancreatic carcinoma were categorized as a unity of clinical disease due to their close anatomical correlation and mutual functional assistance. To verify the universal applicability of the PAAD-IRGS, the TCGA-LIHC data was used to validate the findings. S100A2, S100P and MMP12 were all over expressed in tumor tissues based on paired (*P* < 0.01) (Fig. 8A) and unpaired expression analysis (*P* < 0.001) (Fig. 8B). The diagnostic ROC curves also showed their independent and unified diagnostic value for LIHC (S100P: AUC = 0.739; S100A2: AUC = 0.723; MMP12: AUC = 0.773; model: AUC = 0.812, 95%CI = 0.767–0.857) (Fig. 8C,D). LIHC patients had a worse OS in S100P (HR = 1.43, 95% CI = 1.01–2.02, *P* = 0.44)/S100A2 (HR = 1.81, 95% CI = 1.27–2.57, *P* = 0.001)/MMP12 (HR = 1.58, 95% CI = 1.11–2.23, *P* = 0.01) high-expression group (Fig. 8E) and PAAD-IRGS high-risk group (HR = 1.83, 95% CI = 1.29–2.60, *P* = 0.001) (Fig. 8F). PAAD-IRGS also had a considerable prognostic value for LIHC patients according to ROC analysis (1-year: AUC = 0.651; 2-year: AUC = 0.612; 3-year: AUC = 0.597) (Fig. 8G) and DCA (Fig. 8H). Furthermore, we extracted baseline characteristics of TCGA-LIHC shown in Table 4 and conducted univariate and multivariate COX regression analysis to establish a nomogram based on PAAD-IRGS and multiple clinicopathologic factors (Fig. 8I). T3&T4 stage (*P* < 0.001), M1 stage (*P* = 0.017), pathological stage III&IV (*P* < 0.001), tumor-bearing status (*P* < 0.001) and PAAD-IRGS (*P* < 0.001) were significantly correlated with OS. Tumor-bearing status (HR = 1.992, 95%CI = 1.246–3.185, *P* = 0.004) and PAAD-IRGS (HR = 2.180, 95%CI = 1.180–4.026, *P* = 0.013) were independent factors impacting the OS of patients with LIHC (Table 5). Nomogram calibration curves (Fig. 8J) showed good predictive accuracy of the model, and DCA (Fig. 8K) confirmed the clinical utility of the nomogram. Consistent with the nomogram of PAAD, the comprehensive nomogram of LIHC showed the best accuracy (C-Index = 0.666, 95%CI = 0.630–0.701) than any other indicator (Table 3).

**Table 4:** Baseline characteristics of patients (TCGA-LIHC).

Characteristics	Levels	Low-risk group	High-risk group	*p* value*
		N = 186	N = 187	
T stage, n (%)	T1	106 (57.9%)	77 (41.2%)	**0.011**
	T2	36 (19.7%)	58 (31%)	
	T3	36 (19.7%)	44 (23.5%)	
	T4	5 (2.7%)	8 (4.3%)	
N stage, n (%)	N0	132 (98.5%)	122 (98.4%)	1.000
	N1	2 (1.5%)	2 (1.6%)	
M stage, n (%)	M0	134 (99.3%)	134 (97.8%)	0.622
	M1	1 (0.7%)	3 (2.2%)	
Pathologic stage, n (%)	Stage I	102 (59%)	71 (40.3%)	**0.002**
	Stage II	32 (18.5%)	54 (30.7%)	
	Stage III	38 (22%)	47 (26.7%)	
	Stage IV	1 (0.6%)	4 (2.3%)	
Tumor status, n (%)	Tumor free	104 (57.8%)	98 (56.3%)	0.866
	With tumor	76 (42.2%)	76 (43.7%)	
Gender, n (%)	Female	53 (28.5%)	68 (36.4%)	0.130
	Male	133 (71.5%)	119 (63.6%)	
Age, n (%)	≤ 60	81 (43.5%)	96 (51.3%)	0.161
	> 60	105 (56.5%)	91 (48.7%)	
Residual tumor, n (%)	R0	169 (96.6%)	157 (92.9%)	0.176
	R1	6 (3.4%)	11 (6.5%)	
	R2	0 (0%)	1 (0.6%)	
Histologic grade, n (%)	G1	39 (21.5%)	16 (8.6%)	** < 0.001**
	G2	94 (51.9%)	84 (44.9%)	
	G3	42 (23.2%)	81 (43.3%)	
	G4	6 (3.3%)	6 (3.2%)	
Adjacent hepatic tissue inflammation, n (%)	None	64 (50.8%)	54 (49.1%)	0.966
	Mild	53 (42.1%)	48 (43.6%)	
	Severe	9 (7.1%)	8 (7.3%)	
AFP(ng/ml), n (%)	≤ 400	116 (81.7%)	99 (72.3%)	0.084
	> 400	26 (18.3%)	38 (27.7%)	
Albumin(g/dl), n (%)	< 3.5	40 (25.3%)	29 (20.6%)	0.403
	≥ 3.5	118 (74.7%)	112 (79.4%)	
BMI, n (%)	≤ 25	83 (49.4%)	94 (56%)	0.275
	> 25	85 (50.6%)	74 (44%)	
Child–Pugh grade, n (%)	A	120 (90.9%)	98 (90.7%)	0.902
	B	11 (8.3%)	10 (9.3%)	
	C	1 (0.8%)	0 (0%)	
Fibrosis Ishak score, n (%)	0	44 (38.6%)	31 (31%)	0.607
	1/2	14 (12.3%)	17 (17%)	
	3/4	14 (12.3%)	14 (14%)	
	5/6	42 (36.8%)	38 (38%)	
Vascular invasion, n (%)	No	118 (73.3%)	90 (57.7%)	**0.005**
	Yes	43 (26.7%)	66 (42.3%)	

Data are presented as n (%).

*LIHC* liver hepatocellular carcinoma.

*Compared with each group (Fisher exact test, or Pearson’s chi-square test). *p* value < 0.05 was considered statistically significant (highlighted in bold).

**Table 5:** The univariate and multivariate analysis for the OS (TCGA-LIHC).

Characteristics		Total (N)	Univariate analysis		Multivariate analysis	
			Hazard ratio (95% CI)	*P* value*	Hazard ratio (95% CI)	*P* value*
T stage	T1&T2	277	Reference		NA	
	T3&T4	93	2.598 (1.826–3.697)	** < 0.001**	1.441 (0.196–10.607)	0.720
N stage	N0	254	Reference			
	N1	4	2.029 (0.497–8.281)	0.324		
M stage	M0	268	Reference		NA	
	M1	4	4.077 (1.281–12.973)	**0.017**	1.237 (0.294–5.203)	0.771
Pathologic stage	Stage I	173	Reference		NA	
	Stage II	86	1.416 (0.868–2.312)	0.164	1.425 (0.765–2.653)	0.264
	Stage III&IV	90	2.823 (1.862–4.281)	** < 0.001**	1.793 (0.240–13.410)	0.570
Tumor status	Tumor free	202	Reference		NA	
	With tumor	152	2.317 (1.590–3.376)	** < 0.001**	1.992 (1.246–3.185)	**0.004**
Gender	Female	121	Reference			
	Male	252	0.793 (0.557–1.130)	0.200		
Age	≤ 60	177	Reference			
	> 60	196	1.205 (0.850–1.708)	0.295		
BMI	≤ 25	177	Reference			
	> 25	159	0.798 (0.550–1.158)	0.235		
Residual tumor	R0	326	Reference			
	R1&2	18	1.604 (0.812–3.169)	0.174		
Histologic grade	G1	55	Reference			
	G2	178	1.162 (0.686–1.968)	0.577		
	G3&G4	135	1.222 (0.710–2.103)	0.469		
Adjacent hepatic tissue inflammation	None	118	Reference			
	Mild&Severe	118	1.194 (0.734–1.942)	0.475		
AFP (ng/ml)	≤ 400	215	Reference			
	> 400	64	1.075 (0.658–1.759)	0.772		
Albumin (g/dl)	< 3.5	69	Reference			
	≥ 3.5	230	0.897 (0.549–1.464)	0.662		
Child–Pugh grade	A	218	Reference			
	B&C	22	1.643 (0.811–3.330)	0.168		
Fibrosis Ishak score	0&1/2	106	Reference			
	3/4&5/6	108	0.740 (0.445–1.232)	0.247		
Vascular invasion	No	208	Reference			
	Yes	109	1.344 (0.887–2.035)	0.163		
Model		373	2.718 (1.676–4.410)	** < 0.001**	2.180 (1.180–4.026)	**0.013**

*OS* overall survival, *CI* confidence interval, *NA* reference group or could not be evaluated.

*Compared with each group (log-rank test or omnibus test for univariate, Cox regression analysis with adjusted hazard for multivariate). *p* < 0.05 means statistically significant (highlighted in bold).

**Figure 9:**
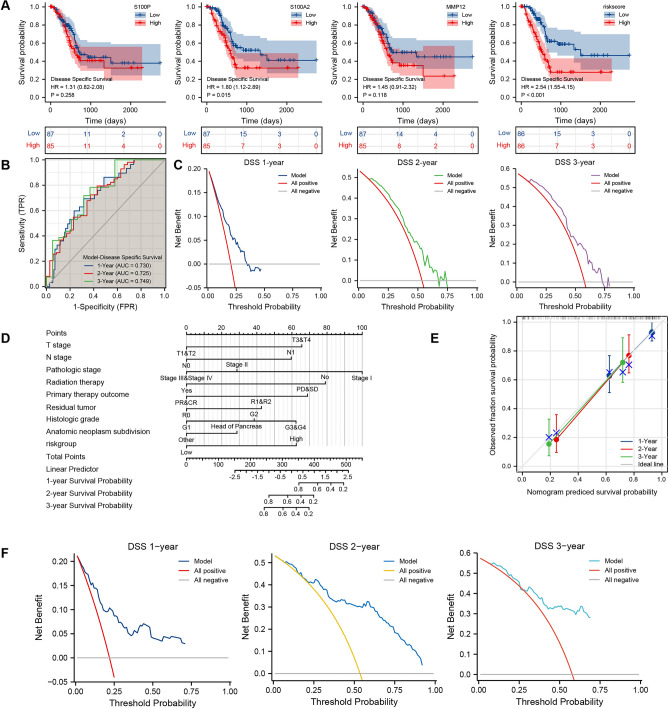
Establishment and assessment of PAAD-IRGS-based nomograms for DSS in TCGA-PAAD. (**A**) Single-gene and IRGs signature Survival analysis of DSS in PAAD; (**B**) the time-dependent ROC curve of the PAAD-IRGS for predicting 1, 2, and 3-year DSS; (**C**) decision curve analysis for evaluating the PAAD-IRGS; (**D**) an PAAD-IRGS-based nomogram included with 8 clinical components predicting 1, 2, and 3-year DSS of PAAD; (**E**) nomogram calibration curve for 1, 2, and 3-year. (**E**) Decision curve analysis for evaluating the net benefits of nomogram at 1, 2, and 3 years. DSS, disease-specific survival; *P < 0.05, **P < 0.01, ***P < 0.001.

**Figure 10:**
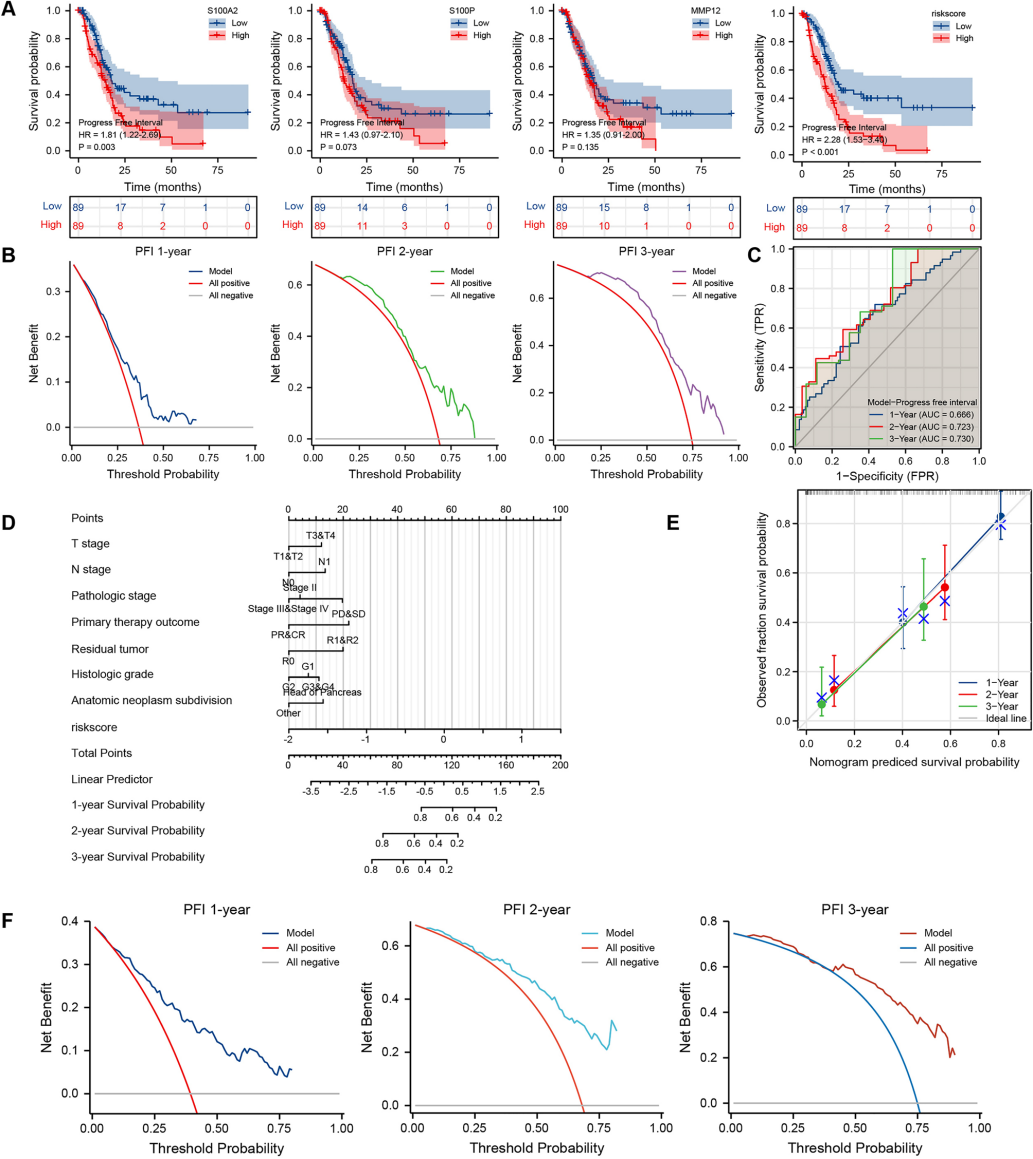
Establishment and assessment of PAAD-IRGS-based nomograms for PFI in TCGA-PAAD. (**A**) Single-gene and PAAD-IRGS survival analysis of PFI in PAAD; (**B**) the time-dependent ROC curve of the PAAD-IRGS for predicting 1, 2, and 3-year PFI; (**C**) decision curve analysis for evaluating the PAAD-IRGS; (**D**) an PAAD-IRGS-based nomogram included with 7 clinical components predicting 1, 2, and 3-year PFI of PAAD; (**E**) nomogram calibration curve for 1, 2, and 3-year. (**E**) Decision curve analysis for evaluating the net benefits of nomogram at 1, 2, and 3 years. *PFI* progress free interval; *P < 0.05, **P < 0.01, ***P < 0.001.

For the further expanded application of PAAD-IRGS, we found that it performed well in predicting disease-specific survival (DSS) and progression-free interval (PFI) of PAAD patients. Patients in PAAD-IRGS high-risk group had a significantly worse DSS (HR = 2.54, 95%CI = 1.55–4.15, *P* < 0.001) (Fig. 9A). Time-dependent ROC showed its robust prognostic predictive value (1-year: AUC = 0.730; 2-year: AUC = 0.724; 3-year: AUC = 0.749) and DCA further validated its clinical applicability (C-Index = 0.680, 95%CI = 0.649–0.711) (Fig. 9B,C). We constructed a comprehensive nomogram composed of PAAD-IRGS and clinicopathological factors (Table 6) (Fig. 9D). Its accuracy and efficiency were evaluated (C-Index = 0.775, 95%CI = 0.742–0.808, Table 3) (Fig. 9E,F). Similarly, Patients in PAAD-IRGS high-risk group had a significantly worse PFI (HR = 2.28, 95%CI = 1.53–3.40, *P* < 0.001) (Fig. 10A). The model had good clinical utility (C-Index = 0.649, 95%CI = 0.618–0.681) (Fig. 10B) and predictive value for prognosis (1-year: AUC = 0.666; 2-year: AUC = 0.723; 3-year: AUC = 0.730) (Fig. 10C). The nomogram based on this model is shown in Fig. 10D using variables summarized in Table 7. The validation analysis results are in Table 3 and Fig. 10E,F (C-Index = 0.742, 95%CI = 0.712–0.771).

**Table 6:** The univariate and multivariate analysis for the DSS (TCGA-PAAD).

Characteristics		Total (N)	Univariate analysis		Multivariate analysis	
			Hazard ratio (95% CI)	*P* value*	Hazard ratio (95% CI)	*P* value*
T stage	T1&T2	30	Reference			
	T3&T4	140	3.119 (1.346–7.229)	**0.008**	3.275 (0.768–13.970)	0.109
N stage	N0	48	Reference			
	N1	119	2.746 (1.473–5.121)	**0.001**	2.787 (1.138–6.825)	**0.025**
M stage	M0	76	Reference			
	M1	5	0.896 (0.212–3.777)	0.881		
Pathologic stage	Stage I	20	Reference			
	Stage II	141	3.294 (1.191–9.110)	**0.022**	0.313 (0.045–2.192)	0.242
	Stage III&IV	8	2.401 (0.530–10.874)	0.256	0.267 (0.025–2.874)	0.276
Gender	Female	76	Reference			
	Male	96	0.751 (0.473–1.194)	0.227		
Age	≤ 65	92	Reference			
	> 65	80	1.067 (0.670–1.701)	0.784		
Radiation therapy	No	114	Reference			
	Yes	43	0.445 (0.238–0.834)	**0.011**	0.346 (0.161–0.742)	**0.006**
Primary therapy outcome	PD&SD	56	Reference			
	PR&CR	79	0.283 (0.164–0.490)	** < 0.001**	0.321 (0.169–0.608)	** < 0.001**
Residual tumor	R0	103	Reference			
	R1&2	55	1.861 (1.137–3.046)	**0.013**	1.934 (1.028–3.639)	**0.041**
Histologic grade	G1	30	Reference		NA	
	G2	92	1.862 (0.889–3.898)	0.099	1.191 (0.414–3.421)	0.746
	G3&G4	48	2.594 (1.199–5.611)	**0.016**	1.585 (0.522–4.809)	0.416
Anatomic neoplasm subdivision	Head of Pancreas	133	Reference			
	Other	39	0.447 (0.234–0.854)	**0.015**	0.883 (0.378–2.062)	0.773
Smoker	No	64	Reference			
	Yes	74	1.075 (0.643–1.799)	0.783		
Alcohol history	No	62	Reference			
	Yes	98	1.211 (0.733–2.002)	0.454		
History of diabetes	No	105	Reference			
	Yes	35	0.820 (0.425–1.581)	0.553		
History of chronic pancreatitis	No	124	Reference			
	Yes	11	0.888 (0.354–2.232)	0.801		
Family history of cancer	No	46	Reference			
	Yes	60	0.994 (0.553–1.784)	0.983		
Model		172	2.718 (1.749–4.226)	** < 0.001**	3.240 (1.656–6.342)	** < 0.001**

*DSS* disease specific survival, *CI* confidence interval, *NA* reference group or could not be evaluated.

*Compared with each group (log-rank test or Omnibus test for univariate, Cox regression analysis with adjusted hazard for multivariate). *p* < 0.05 means statistically significant (highlighted in bold).

**Table 7:** The univariate and multivariate analysis for the PFI (TCGA-PAAD).

Characteristics		Total (N)	Univariate analysis		Multivariate analysis	
			Hazard ratio (95% CI)	*P* value*	Hazard ratio (95% CI)	*P* value*
T stage	T1&T2	31	Reference			
	T3&T4	145	2.414 (1.309–4.452)	**0.005**	1.536 (0.605–3.903)	0.367
N stage	N0	50	Reference			
	N1	123	1.735 (1.113–2.705)	**0.015**	1.613 (0.853–3.048)	0.141
M stage	M0	79	Reference			
	M1	5	0.837 (0.300–2.336)	0.734		
Pathologic stage	Stage I	21	Reference			
	Stage II	146	2.966 (1.353–6.501)	**0.007**	0.572 (0.152–2.162)	0.410
	Stage III&IV	8	2.965 (0.976–9.014)	0.055	0.494 (0.101–2.422)	0.384
Gender	Female	80	Reference			
	Male	98	0.968 (0.658–1.423)	0.867		
Age	≤ 65	93	Reference			
	> 65	85	1.256 (0.848–1.861)	0.256		
Radiation therapy	No	118	Reference			
	Yes	45	0.744 (0.474–1.168)	0.199		
Primary therapy outcome	PD&SD	58	Reference			
	PR&CR	81	0.336 (0.216–0.524)	** < 0.001**	0.454 (0.278–0.743)	**0.002**
Residual tumor	R0	107	Reference			
	R1&2	57	2.253 (1.494–3.398)	** < 0.001**	2.042 (1.239–3.363)	**0.005**
Histologic grade	G1	31	Reference		NA	
	G2	95	1.740 (0.956–3.170)	0.070	0.774 (0.355–1.685)	0.518
	G3&G4	50	2.570 (1.361–4.853)	**0.004**	1.149 (0.500–2.641)	0.743
Anatomic neoplasm subdivision	Head of Pancreas	138	Reference			
	Other	40	0.495 (0.299–0.820)	**0.006**	0.637 (0.335–1.210)	0.168
Smoker	No	65	Reference			
	Yes	79	1.048 (0.683–1.606)	0.831		
Alcohol history	No	65	Reference			
	Yes	101	1.217 (0.799–1.851)	0.360		
History of diabetes	No	108	Reference			
	Yes	38	0.783 (0.460–1.333)	0.368		
History of chronic pancreatitis	No	128	Reference			
	Yes	13	0.885 (0.426–1.840)	0.744		
Family history of cancer	No	47	Reference			
	Yes	63	0.955 (0.574–1.590)	0.860		
Model		178	2.718 (1.881–3.929)	** < 0.001**	2.783 (1.670–4.639)	** < 0.001**

*PFI* progress free interval, *CI* confidence interval, *NA* reference group or could not be evaluated.

*Compared with each group (log-rank test or Omnibus test for univariate, Cox regression analysis with adjusted hazard for multivariate). *p* < 0.05 means statistically significant (highlighted in bold).

### Immunity associated analysis of PAAD-IRGS

**Figure 11:**
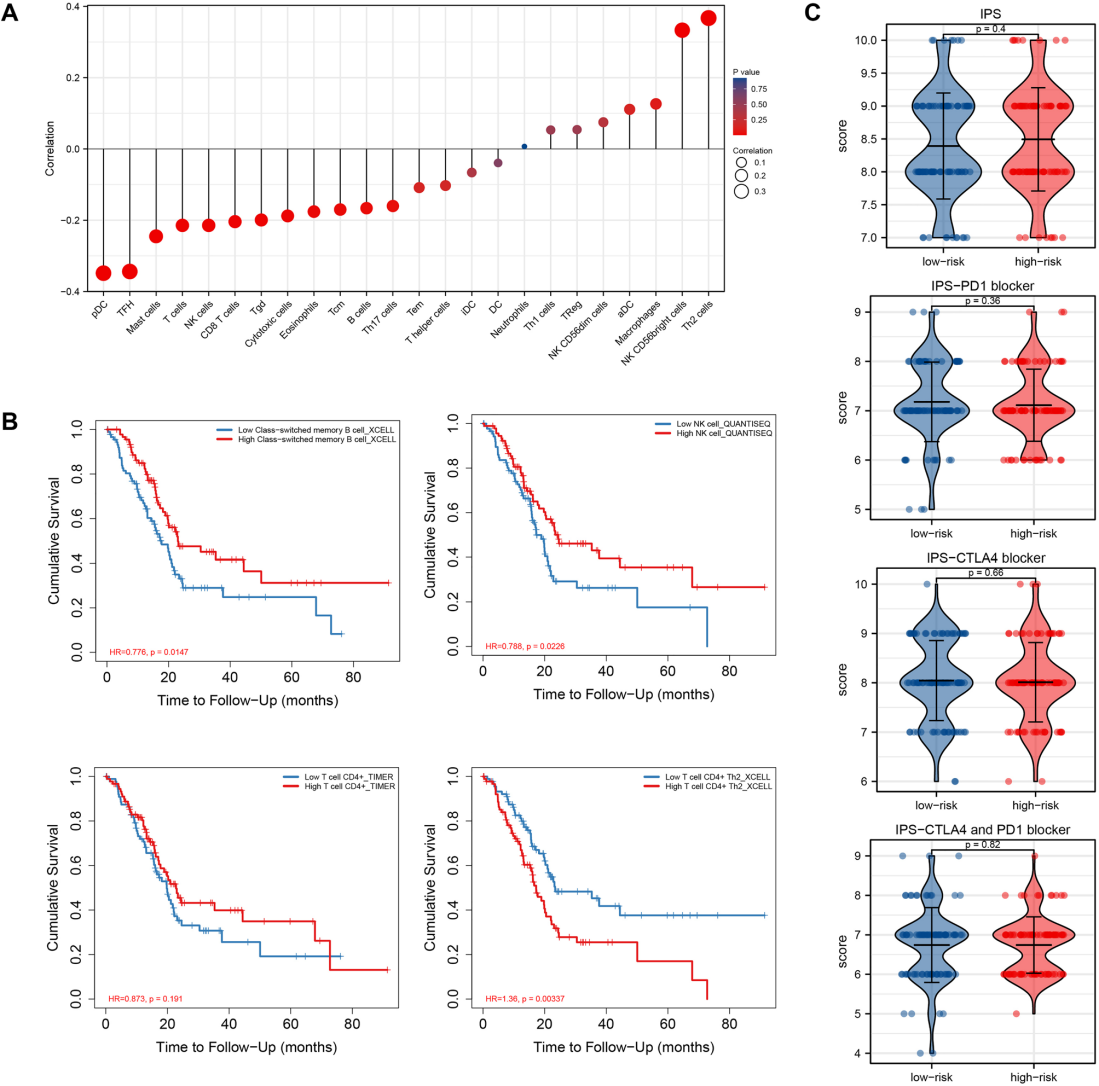
Analysis of correlation between PAAD-IRGS and immune-related cells and relevant immunotherapy. (**A**) Lollipop plot of PAAD-IRGS and immune infiltration cells correlation in TCGA-PAAD; (**B**) survival analysis of immune-related cells infiltration in PAAD; (**C**) analysis of immunotherapeutic efficiency based on PAAD-IRGS in TCGA-PAAD. *IPS* immunephenoscore. *P < 0.05, **P < 0.01, ***P < 0.001.

Tumor-infiltrating immunocytes (TIICs) play an important role in the complex tumor-immune microenvironment and have been shown to influence the progression of various tumors^49,50^. Thus, we must investigate any relationship between PAAD-IRGS and TIICs in PAAD. We used a lollipop plot to perform the correlation analysis of 24 immune-related cells (Fig. 11A). There was a significant positive correlation between PAAD-IRGS and NK CD56bright cells (r = 0.333, *P* < 0.001) and Th2 cells (r = 0.367, *P* < 0.001) and negative correlation with plasmacytoid dendritic cells (pDC) (r = −0.348, *P* < 0.001) and follicular helper T cell (TFH) (r = -0.344, *P* < 0.001). However, only B cell, CD4+ T cell and NK cell infiltration levels were correlated with OS of PAAD patients. Patients with high B cell (HR = 0.776, *P* = 0.0147) or NK cell (HR = 0.788, *P* = 0.0226) infiltration level had a better OS, while high CD4+ T cell + Th2 cell infiltration level associated with worse OS (HR = 1.36, *P* = 0.00337) (Fig. 11B). There was no statistically significant difference between high-risk and low-risk groups in patients with PD-1 blocker/CTLA4 blocker/CTLA4&PD-1 blocker or without immune-blocker (Fig. 11C).

**Figure 12:**
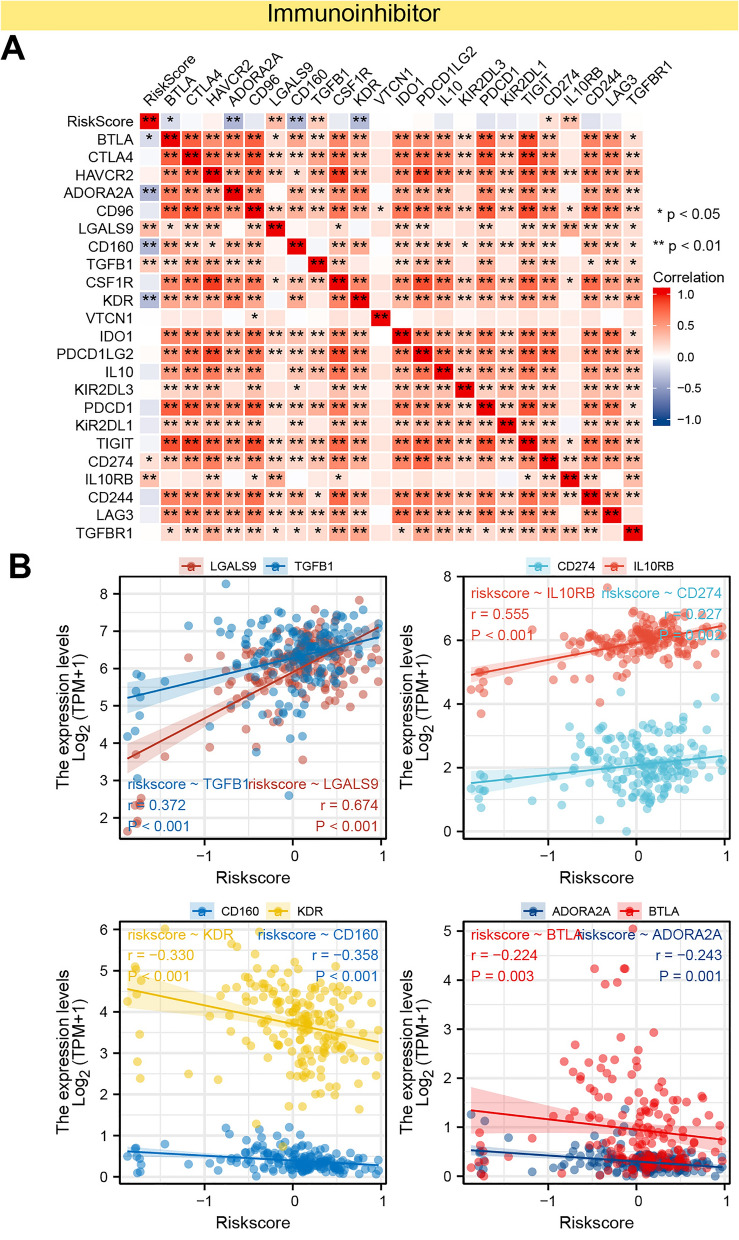
Analysis of correlation between PAAD-IRGS and immuno-inhibitors. (**A**) Immune-inhibitor genes—PAAD-IRGS heatmap; (**B**) correlation analysis of immune-inhibitor genes and PAAD-IRGS. *P < 0.05, **P < 0.01, ***P < 0.001.

**Figure 13:**
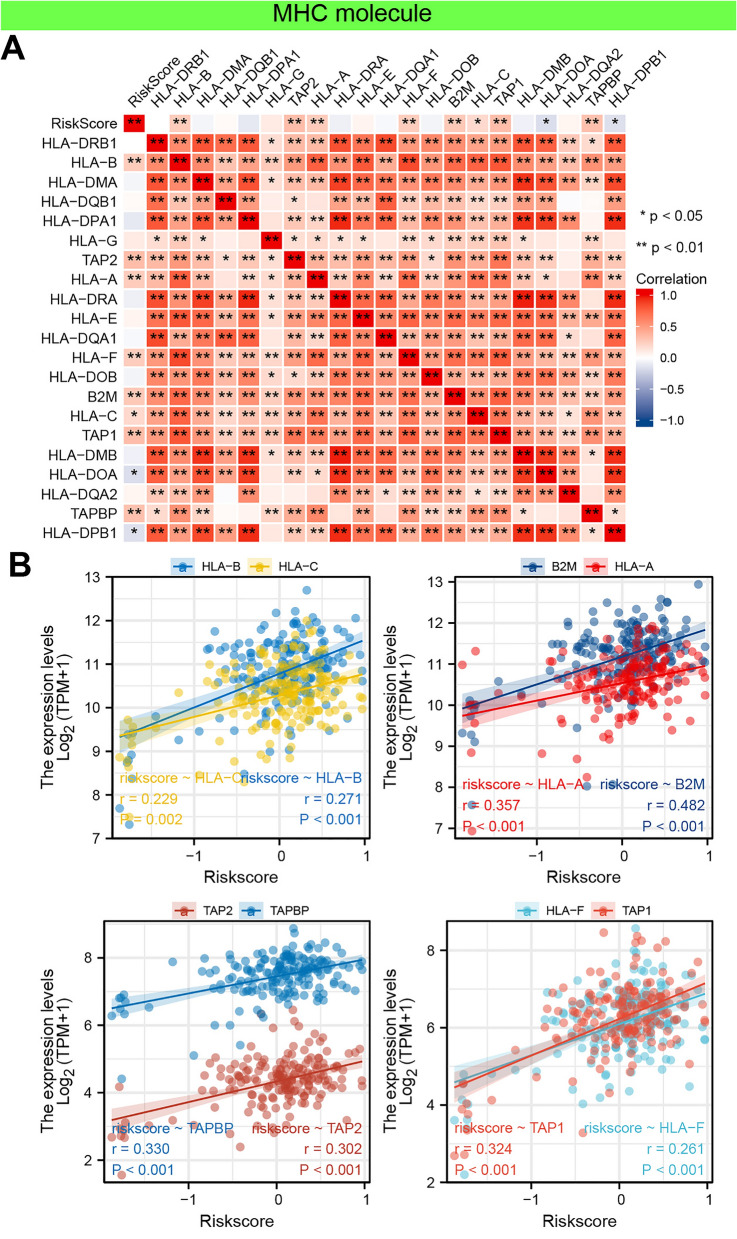
Analysis of correlation between PAAD-IRGS and MHC molecule. (**A**) MHC molecule genes—PAAD-IRGS heatmap; (**B**) correlation analysis of MHC molecule genes and PAAD-IRGS. *MHC* major histocompatibility complex; *P < 0.05, **P < 0.01, ***P < 0.001.

**Figure 14:**
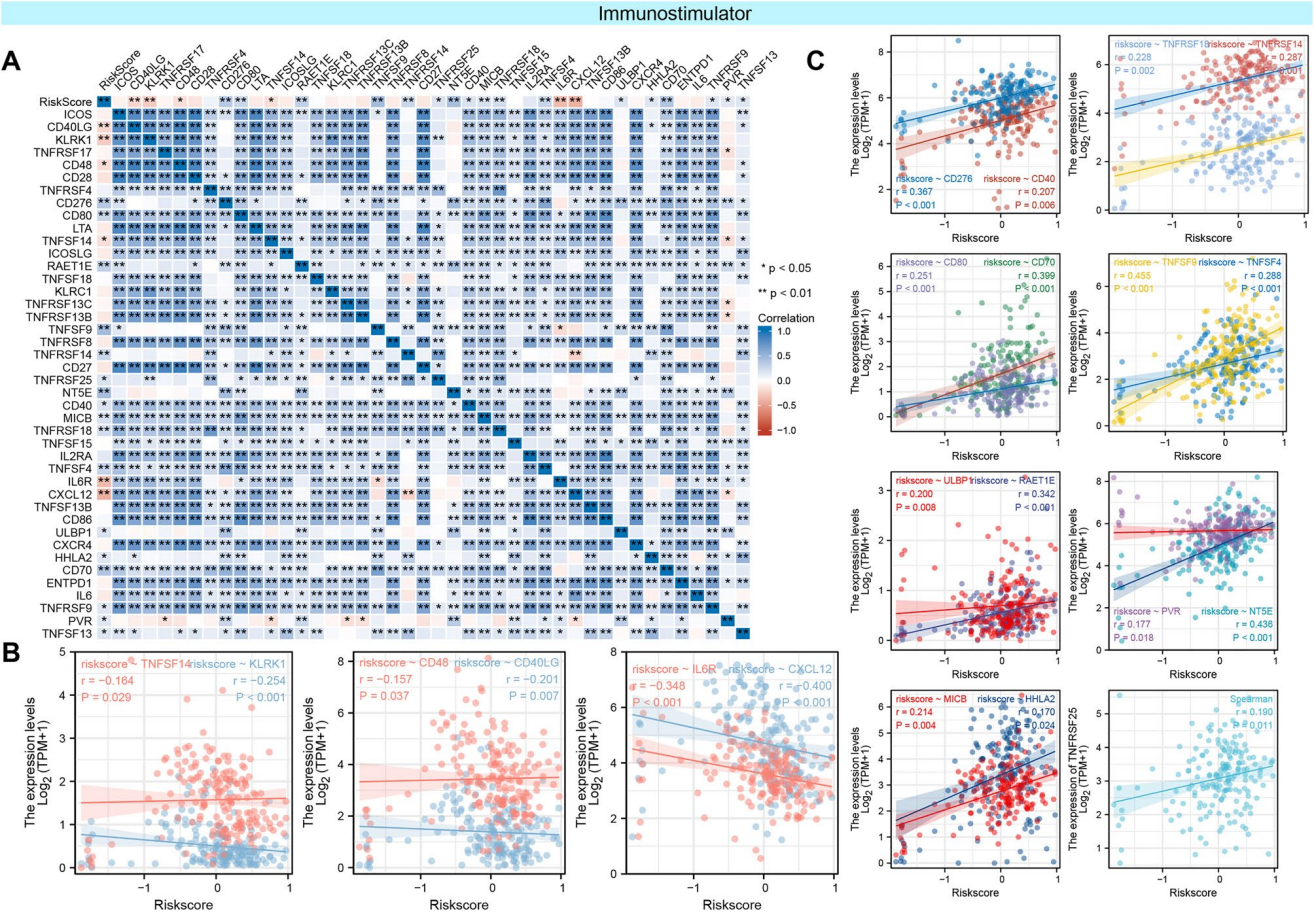
Analysis of correlation between PAAD-IRGS and immuno-stimulators. (**A**) Immune-stimulator genes—PAAD-IRGS e heatmap; (**B**) correlation analysis of immune-stimulator genes and PAAD-IRGS (negative). (**C**) Correlation analysis of immune-stimulator genes and PAAD-IRGS (positive). *P < 0.05, **P < 0.01, ***P < 0.001.

As a supplement, we conducted correlation analysis between immunomodulators and PAAD-IRGS, which were visualized as heatmaps (Figs. 12A, 13A, 14A). For immune-inhibitors, PAAD-IRGS had positive correlation with TGFB1 (r = 0.372, *P* < 0.001), LGALS9 (r = 0.674, *P* < 0.001), IL10RB (r = 0.555, *P* < 0.001) and CD274 (r = 0.227, *P* = 0.002), negative correlation with KDR (r = −0.330, *P* < 0.001), CD160 (r = −0.358, *P* < 0.001), BTLA (r = −0.224, *P* = 0.003) and ADORA2A (r = −0.243, *P* = 0.001) (Fig. 12B). For (MHC) molecule, HLA-B (r = 0.271, *P* < 0.001), HLA-C (r = 0.229, *P* = 0.002), B2M (r = 0.482, *P* < 0.001), HLA-A (r = 0.357, *P* < 0.001), TAP2 (r = 0.302, *P* < 0.001), TAPBP (r = 0.330, *P* < 0.001), HLA-F (r = 0.261, *P* < 0.001) and TAP1 (r = 0.324, *P* < 0.001) were positively related with PAAD-IRGS (Fig. 13B). As to immune-stimulators, there were 6 genes negatively related with PAAD-IRGS (Fig. 14B) while 15 genes had a positive correlation (Fig. 14C).

### PAAD-IRGS related drugs

**Figure 15:**
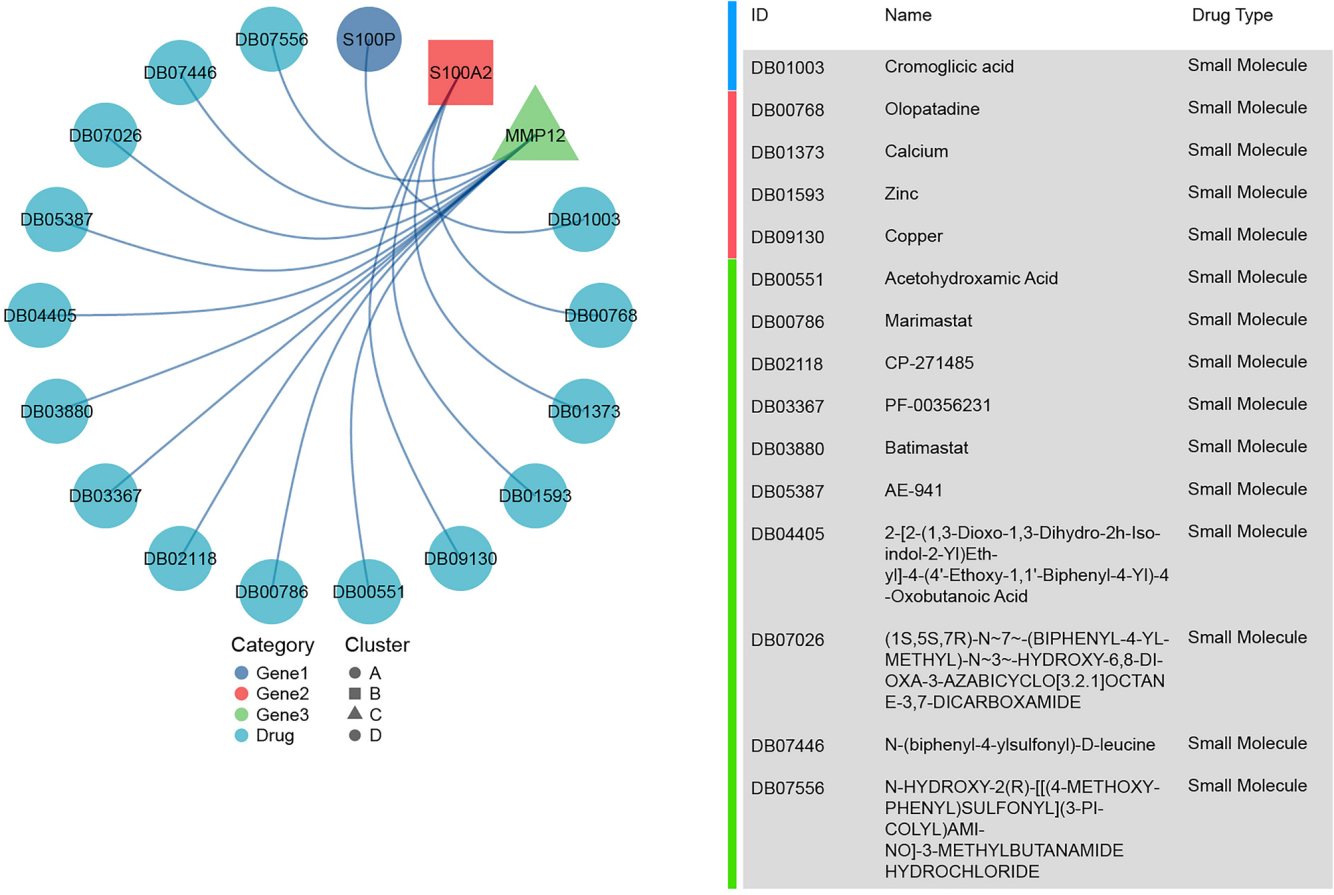
Prediction of PAAD-IRGS associated drugs. The network plot showed several potential drugs targeted with the IRGs.

TISIDB is a web portal for tumor and immune system interaction, which supports genomics, transcriptomics, and clinical data from TCGA and mechanism, and drug information from public databases. We can only obtain potential drugs associated with PAAD-IRGS, which is demonstrated in a network diagram (Fig. 15). Currently, drugs targeting PAAD-IRGS (S100P, S100A2 and MMP12) remained in the experimental stage, and effective targeted drugs for pancreatic cancer are still in the blank.

### Analysis of protein expression of the PAAD-IRGS

We obtained the protein expression pattern of S100P and S100A2 in different cancers based on the HPA database. Expression of S100P in most pancreatic (83.3%) and liver (54.5%) cancers showed moderate to intense cytoplasmic and nuclear staining (Fig.S1A). Immunohistochemistry (IHC) results also confirmed that S100P was highly expressed in PAAD and LIHC than in corresponding normal tissues (Fig.S1B). Although the level of S100A2 protein expression was lower than that of S100P (Fig.S2A), we can still observe the moderate intensity of S100A2 in PAAD and LIHC than in corresponding normal tissues (Fig.S2B). The information on MMP12 in the HPA database was absent, we conducted further verification using the UALCAN database. To be consistent, the protein expression of S100P was higher in PAAD and LIHC than in corresponding normal tissues (*P* < 0.001) (Fig.S3A), as well as in MMP12 (*P* < 0.001) (Fig.S3B). Despite the data absent in LIHC, the protein expression of S100A2 was higher in PAAD than in normal tissues (*P* < 0.01) (Fig.S3C).

## Discussions

Although pancreatic cancer is still one of the leading causes of cancer-related death worldwide, some improvements in patient outcomes have been made due to advancements in therapeutics^51^. Since there are no obvious clinical symptoms in the early stage, pancreatic cancer is usually advanced at diagnosis. Secondly, the high mortality of PAAD seems to be inextricably associated with its suppressed immune microenvironment and significant decrease of T cell infiltration levels in the tumor^52^. Although immunotherapy has revolutionized the cancer treatment model, PAAD patients rarely respond to these therapies due to poor activation and infiltration of T cells in the tumor-immunity microenvironment (TIME). Recent research has revealed potential epigenetic-transcriptional mechanisms by which tumor cells remodel their TIME and suggested EGFR inhibitors as potential immunotherapy sensitizers in PAAD^53^. Intra-tumoral IFN-γ-producing Th22 cells were reported to be associated with TNM staging and the worst outcomes in PAAD^54^. γδ T Cells were also considered to promote pancreatic oncogenesis by restraining αβ T Cells activation^55^. Each T cell subpopulations secretes different cytokines and chemokines that modulate the immune response in synergistic and opposite ways^56^. Additionally, expansion of immunosuppressive B cells induced by IL-1β might promote PAAD^57^, and many extracellular matrix (ECM) components, including collagen, growth factors, cytokines, chemokines, and cancer-associated fibroblast (CAF) play vital role in tumor progression^58^. All tumor-immunity components in the TIME interact continuously, constructing a complex stroma-tumor crosstalk network. Due to the complexity of tumor-immunity mechanisms, there is still no effective way to predict prognosis in clinical practice. Our study aimed to discover immune-related biomarkers and establish a robust model to predict prognosis in PAAD patients.

The TCGA-PAAD dataset was used to screen for potentially immune-related DEGs , then analyzed for differential expression and intersection. GO and KEGG enrichment analyses were also performed to confirm that the mechanisms involved in these genes were focused on immune-related pathways (Fig. 2D). Furthermore, we narrowed down the results by Lasso regression analysis and obtained three key IRGs finally through the univariate and multivariate Cox regression analysis. The PAAD-IRGS comprised of S100P, S100A2 and MMP12 had an outstanding diagnostic value (Fig. 4D) and accurately predicted the prognosis for PAAD patients (Fig. 5A,B). We specially performed secondary enrichment analysis on PAAD-IRGS, revealing that this model was also associated with pathways of ECM and cell-membrane junction and immune-related pathways (Fig. 4C).

Among the three genes, S100P, a 95-amino-acid protein belonging to the S100 family, was regarded as a promising diagnostic^59^ and prognostic biomarker^60^ for pancreatic cancer with a potential mechanism of regulating invasion into the lymphatic endothelial monolayer^61^, which is consistent with our results. S100A2, another member of the S100 family, was reported as a prognostic biomarker involved in immune infiltration and immunotherapy response prediction in pancreatic cancer^62^, which matches our findings. Turn to MMP12, as one of the members of the matrix metalloproteinases family, it encodes extracellular matrix participating in the (EMT) which was identified as a strictly programmed shift playing a crucial role in tumor invasion and metastasis^63^. MMP12 was also revealed to be a potential diagnostic biomarker for pancreatic carcinoma. Its up-regulation was associated with a poor prognosis^64^. These genes were verified to be closely correlated with different cancers, especially the diagnosis and prognosis of PAAD, a finding we also made in this work. Although many types of diagnostic or prognostic biomarkers, and even to some extent predictive models have been identified in recent studies^60,65–68^, we discovered three IRGs with high specificity. We integrated them to establish a novel prognostic model for PAAD. Compared with other models, our model had an extremely remarkable performance on both diagnosis and prognosis prediction in PAAD patients.

In our study, patients in the PAAD-IRGS high-risk group had a significantly worse OS than those in the low-risk group (Fig. 4F), indicating that the PAAD-IRGS score may be an independent risk factor when evaluating the prognosis of PAAD patients. Additionally, time-dependent ROC and DCA results (Fig. 5A,B) showed that PAAD-IRGS had a good performance in prediction prognosis. The nomogram integrating PAAD-IRGS and multiple clinicopathological variables showed better accuracy and reliability than any singular variable (Table 3).

We not only evaluated and validated the PAAD-IRGS by using two datasets of pancreatic cancer from GEO, but also investigated its application to hepatocellular carcinoma. Hepatobiliary and pancreatic diseases are often classified into the same category since they are anatomically and functionally linked. Although the cholangiocarcinoma dataset of TCGA was discarded due to its small sample size, we found that the PAAD-IRGS had excellent diagnostic and prognostic value on LIHC patients. We also combined relevant clinicopathological variables with PAAD-IRGS to construct a comprehensive nomogram model, which showed good accuracy and robustness. Based on the results, we might speculate whether the three genes participated in the oncogenesis, progression and metastasis of LIHC and PAAD partially or collectively. However this needs further exploration. We also looked into using PAAD-IRGS to predict DSS and PFI in patients with PAAD. The results of PAAD-IRGS and the relevant prognostic model were encouraging. Unlike other biomarkers that only had diagnostic value, PAAD-IRGS had the dual capability to predict diagnosis and prognosis with high accuracy. Several multiple-genes prognostic model have been established and reported^67,68^. Compared with them, our model had outstanding general applicability with high accuracy and stability. As to the miRNA or lncRNA-related signatures^65,69–71^, our PAAD-IRGS was more stable and convinced; Compared with multiple-gene signatures^9,68^, necroptosis-related gene signature^72^ and m6A-related gene signature^73,74^, which had been reported, our PAAD-IRGS was new and more versatile with outstanding performance. It can be well applied to prognostic prediction of multiple cancers with different prognostic parameters. Its good diagnostic ability for various cancer and its relationship with tumor-immunity would make it promising for further research.

There were several limitations of this research to be concerned about. The limitation to this study worth noting include: there may be an effect on the result due to batch effect and differences in sample sizes that are difficult to eliminate completely. Secondly, although the prognostic value of the PAAD-IRGS was evaluated in multiple datasets, large-scale clinical research is still necessary for further validation. Thirdly, we conducted correlation analyses between PAAD-IRGS and immune-related cells/immunomodulators and disclosed some potential immune-related targets. However, the underlying mechanisms and pathways need further investigation and experiment validation.

In conclusion, our study established a novel prognostic model comprised of three genes with high specificity for predicting prognosis in patients with PAAD. This model demonstrated excellent performance in predicting both diagnosis and prognosis. Since PAAD-IRGS can be generalized, it may be a beneficial predictive model in clinical practice.

## Supplementary Information

Supplementary Information.

Supplementary Figure S1.

Supplementary Figure S2.

Supplementary Figure S3.

Supplementary Legends.

## Data Availability

The original contributions presented in the study are included in the article/supplementary material. Further inquiries can be directed to the corresponding author/s. All data and original files in our work are freely available under a ‘Creative Commons BY 4.0’ license. All methods were carried out in accordance with relevant guidelines and regulations.
