# Supplementary figures and images for: Pancreatic adenocarcinoma associated immune-gene signature as a novo risk factor for clinical prognosis prediction in hepatocellular carcinoma
Source: Sci Rep. 2022 Jul 13;12:11944. doi: 10.1038/s41598-022-16155-w (PMC9279485; doi:10.1038/s41598-022-16155-w)

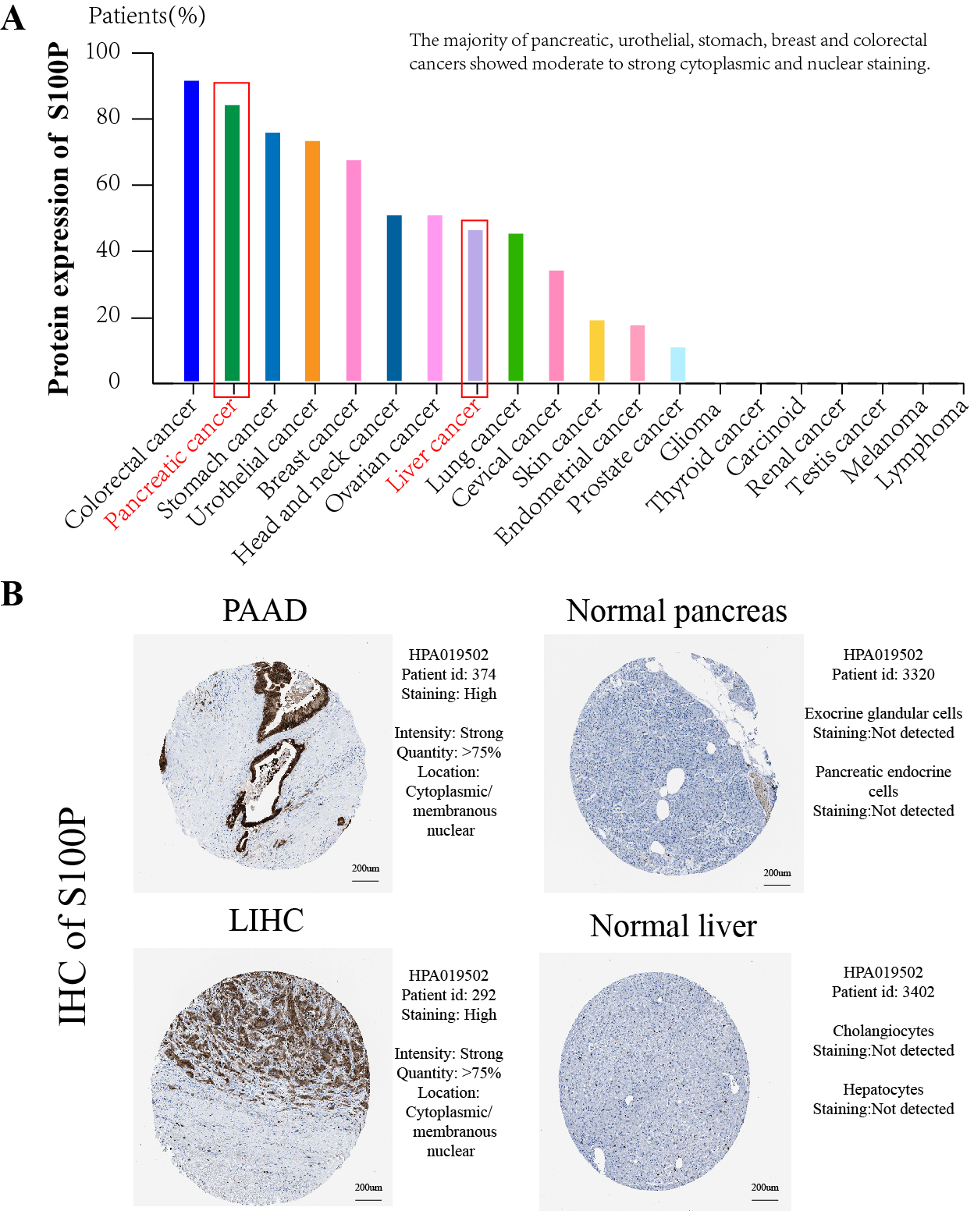

Supplement: Supplementary file 2 — Supplementary Figure S1. [file 41598_2022_16155_MOESM2_ESM.jpg]

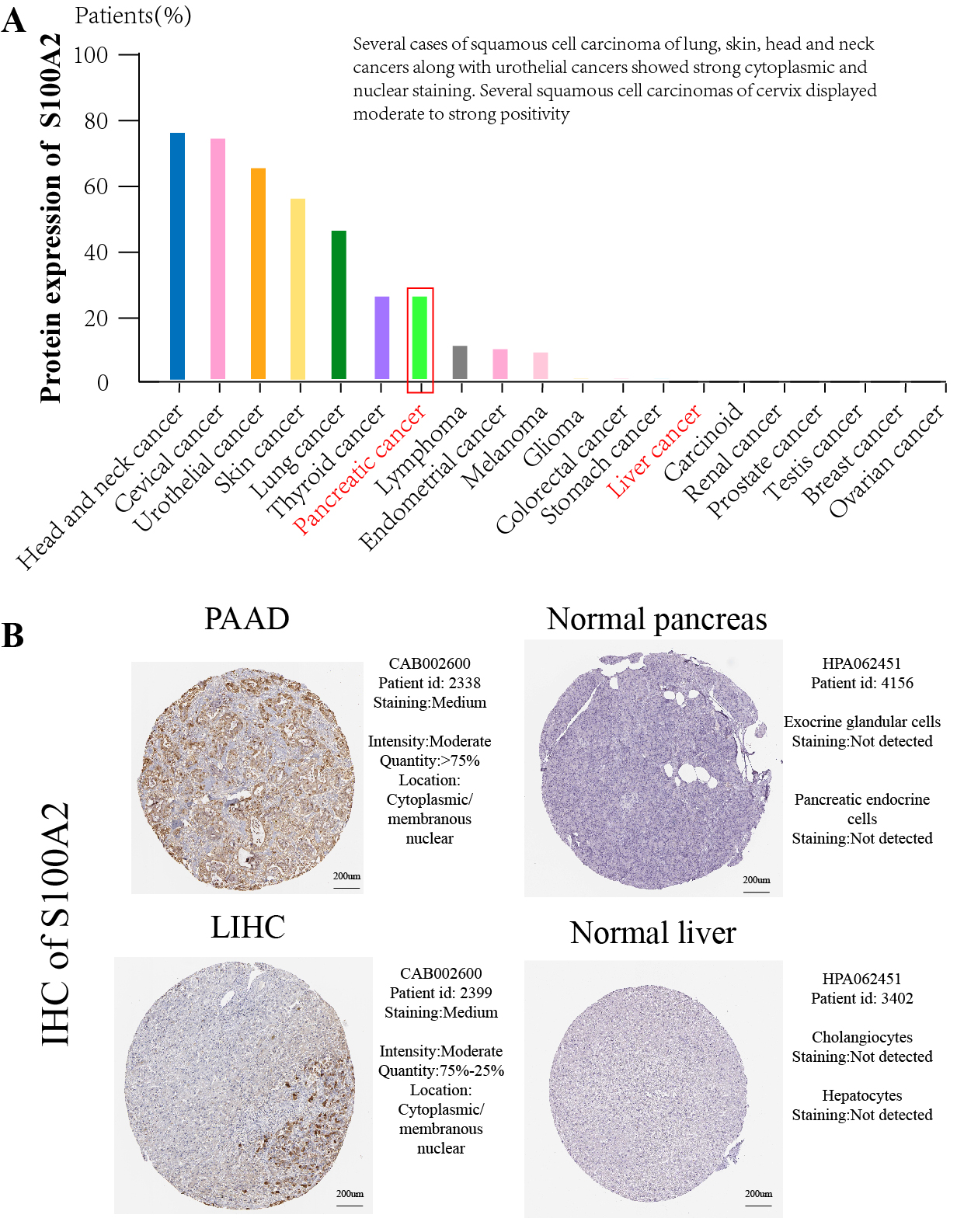

Supplement: Supplementary file 3 — Supplementary Figure S2. [file 41598_2022_16155_MOESM3_ESM.jpg]
