# Supplementary material for: Pancreatic adenocarcinoma associated immune-gene signature as a novo risk factor for clinical prognosis prediction in hepatocellular carcinoma
Source: Sci Rep. 2022 Jul 13;12:11944. doi: 10.1038/s41598-022-16155-w (PMC9279485; doi:10.1038/s41598-022-16155-w)
Supplement: Supplementary file 5 — Supplementary Legends. [file 41598_2022_16155_MOESM5_ESM.docx]

# Supplementary figure legends

**Figure S1. Protein expression of S100P from HPA database.**

(A) Protein expression of S100P among 20 tumor tissues; (B) IHC of S100P between tumor (PAAD/LIHC) and normal tissues. IHC, immunohistochemistry; PAAD, pancreatic adenocarcinoma; LIHC, liver hepatocellular carcinoma.

**Figure S2. Protein expression of S100A2 from HPA database.**

(A) Protein expression of S100A2 among 20 tumor tissues; (B) IHC of S100A2 between tumor (PAAD/LIHC) and normal tissues. IHC, immunohistochemistry; PAAD, pancreatic adenocarcinoma; LIHC, liver hepatocellular carcinoma.

**Figure S3. Protein expression analysis of S100P/S100A2/MMP12 from UALCAN database.**

(A) Protein expression of S100P is higher in PAAD and LIHC, compared with normal tissues; (B) Protein expression of MMP12 is higher in PAAD and LIHC, compared with normal tissues; (C) Protein expression of S100A2 is higher in PAAD, compared with normal tissues; **P* < 0.05, ***P* < 0.01, ****P* < 0.001.
